# *Chlamydia trachomatis* Inc Ct226 is vital for FLI1 and LRRF1 recruitment to the chlamydial inclusion

**DOI:** 10.1128/msphere.00473-24

**Published:** 2024-10-15

**Authors:** Natalie A. Sturd, Lindsey A. Knight, Macy G. Wood, Legacy Durham, Scot P. Ouellette, Elizabeth A. Rucks

**Affiliations:** 1 Department of Pathology, Microbiology, and Immunology, College of Medicine, University of Nebraska Medical Center, Omaha, Nebraska, USA; The University of Texas Medical Branch at Galveston, Galveston, Texas, USA

**Keywords:** FLI1, LRRF1, *Chlamydia trachomatis*, inclusion membrane protein

## Abstract

The obligate intracellular pathogen, *Chlamydia trachomatis*, establishes an intracellular niche within a host membrane-derived vacuole called the chlamydial inclusion. From within this inclusion, *C. trachomatis* orchestrates numerous host-pathogen interactions, in part, by utilizing a family of type III secreted effectors, termed inclusion membrane proteins (Incs). Incs are embedded within the inclusion membrane, and some function to recruit host proteins to the inclusion. Two such recruited host proteins are leucine rich repeat Flightless-1 interacting protein 1 (LRRF1/LRRFIP1) and its binding partner Flightless 1 (FLI1/FLII). Previously, LRRF1 has been shown to interact with Inc protein Ct226/CTL0478. This is the first study to examine interactions of FLI1 with candidate Incs or with LRRF1 during infection. We hypothesized that FLI1 recruitment to the inclusion would be dependent on LRRF1 localization. We demonstrated that FLI1 co-immunoprecipitated with Ct226 but only in the presence of LRRF1. Furthermore, FLI1 localized to the inclusion when LRRF1 was depleted via small interfering RNA, suggesting that FLI1 may have an alternative recruitment mechanism. We further developed a series of CRISPRi knockdown and complementation strains in *C. trachomatis* serovar L2 targeting ct226 and co-transcribed candidate Incs, ct225 and ct224. Simultaneous knockdown of ct226, ct225, and ct224 prevented localization of both FLI1 and LRRF1 to the inclusion, and only complementation of ct226 restored their localization. Thus, we demonstrated Ct226 is critical for FLI1 and LRRF1 localization to the inclusion. Our results also indicate an LRRF1-independent localization mechanism for FLI1, which likely influence their mechanism(s) of action during chlamydial infection.

**IMPORTANCE:**

*Chlamydia trachomatis* is a leading cause of both bacterial sexually transmitted infections and preventable infectious blindness worldwide. As an obligate intracellular pathogen, *C. trachomatis* has evolved multiple ways of manipulating the host to establish a successful infection. As such, it is important to understand host-chlamydial protein-protein interactions as these reveal strategies that *C. trachomatis* uses to shape its intracellular environment. This study looks in detail at interactions of two host proteins, FLI1 and LRRF1, during chlamydial infection. Importantly, the series of CRISPR inference knockdown and complement strains developed in this study suggest these proteins have both independent and overlapping mechanisms for localization, which ultimately will dictate how these proteins function during chlamydial infection.

## INTRODUCTION

In the year 2020, the World Health Organization reported 129 million new *Chlamydia trachomatis* infections worldwide with global infection rates rising steadily since 2013 (1). In the USA alone, direct medical costs of treating infections exceeds 650 million dollars, second only to direct medical costs of human papillomavirus (HPV) and HIV (2). Clinical manifestations of *C. trachomatis* infections are serovar-dependent, as serovars dictate tissue tropism. *C. trachomatis* is capable of infecting multiple host tissues, including the conjunctiva of the eye (serovars A–C), the male and female reproductive tracts (serovars D–K), and the pelvic lymph nodes, (serovars L1–L3), with the L serovars causing lymphogranuloma venereum (3, 4). Importantly, studies estimate upward of 70%–80% of infected women and 50%–60% of infected men are asymptomatic (5, 6), which often results in a delay of treatment and resolution of infection. Chronic infection in the female reproductive tract increases the risk of the infection ascending into the uterus and fallopian tubes, leading to pelvic inflammatory disease (7). Chronic and repeat chlamydial infections increase the risk of uterine and tubal scarring, which have the potential to progress to tubal factor infertility or hospitalization for ectopic pregnancy (7–10). The host-pathogen interactions responsible for the development of host tissue pathology are not fully understood.

*C. trachomatis* is a gram-negative, obligate intracellular pathogen that requires infection of eukaryotic cells to complete its developmental cycle. It alternates between two morphologically and functionally distinct forms: the infectious, non-replicative elementary body (EB) and the non-infectious, replicative reticulate body (RB) (11, 12). The developmental cycle begins with entry of the EB via host-mediated endocytosis, where it resides in a host membrane-derived vacuole. Following entry, the EB rapidly undergoes primary differentiation to an RB, which initiates replication within this pathogen-specific vacuole, termed the chlamydial inclusion (13). Later in development, RBs undergo secondary differentiation to EBs (12), which egress either by host cell lysis or by extrusion to infect new host cells (14). Gene expression throughout the developmental cycle is temporally regulated, and gene transcription profiles are categorized by peak expression into early, mid, and late-cycle development (15, 16). Throughout the developmental cycle, *C. trachomatis* must maintain and grow the inclusion, acquire nutrients, and limit detection by the host until it escapes from the cell. All these require complex and tightly regulated interactions with host proteins and associated signaling pathways while remaining sequestered within the inclusion.

*C. trachomatis* uses a type III secretion system to secrete effectors to establish and maintain their intracellular niche (17, 18). One such family of secreted effectors is known as inclusion membrane proteins, or Incs, which localize within the inclusion membrane (19). Incs are characterized as having at least two transmembrane domains with the N- and C-termini of the protein oriented toward the host cell cytoplasm (20). While some Incs likely support the structural integrity and shape of the inclusion membrane, most studies characterizing Inc function have focused on interactions between Incs and various host proteins (21–25). These Inc-host interactions allow for selective interaction with host cell vesicular compartments, acquisition of host-derived lipids, and avoidance of innate immune defenses (26, 27). However, the full scope of Inc-host and/or Inc-Inc interactions and their impact on host pathways remains unknown.

Previously, we used APEX2 proximity labeling to determine the host protein interactome around the inclusion (28, 29). Two statistically significant hits were the eukaryotic protein leucine rich repeat Flightless-1 interacting protein 1 (LRRF1**/**LRRFIP1/GCF2) and its binding partner, Flightless-1 (FLI1/FLII), both of which localize to the inclusion membrane during chlamydial infection (28). Furthermore, LRRF1 localizes to the inclusion during early mid-cycle development (~12 hours post infection [hpi]) and interacts with the chlamydial Inc protein Ct226/CTL0478 (see Materials and Methods for note on nomenclature) (28). FLI1 and LRRF1 have a multitude of reported functions in uninfected eukaryotic cells. Both are reported to act as regulators of eukaryotic gene expression; FLI1 is a co-activator of nuclear receptor-mediated transcription (e.g., estrogen receptor and glucocorticoid receptor) (30–32), while LRRF1 is a known transcriptional repressor with DNA binding activity (33–36). Both are also involved in regulating host innate immune responses to infection, though their activity has been shown to be both antagonistic and synergistic (37–43). Finally, both proteins are involved in modulation of the actin cytoskeleton. FLI1 binds and prevents actin polymerization and is a prominent negative regulator of fibroblast migration and wound healing (44–50). In contrast, knockdown of LRRF1 reduces migration, cytoskeletal remodeling, and RhoA activation, potentially through its ability to interact with the RhoA-activator LARG (45, 51). Considering the respective activities of FLI1 and LRRF1, it is intriguing that they localize to the inclusion membrane during *C. trachomatis* infection. Given the diversity of independent and collaborative activities of FLI1 and LRFF1, it has been challenging to identify an apparent phenotype associated with their recruitment to the chlamydial inclusion. However, their function during chlamydial infection is likely linked to their mechanism of localization to the inclusion.

For the first time, in this study, we investigate the dynamics of FLI1 localization to the chlamydial inclusion membrane throughout the developmental cycle and interrogate the Inc-host interactions required for both FLI1 and LRRF1 localization. Our data demonstrate that FLI1 interacts with Ct226 in a complex only in the presence of LRRF1 but cannot bind Ct226 independently of LRRF1. Furthermore, we show FLI1 localizes to the chlamydial inclusion independently of LRRF1, potentially indicating an LRRF1-/Ct226-independent mechanism of FLI1 localization. To better understand FLI1 and LRRF1 localization dynamics, we used a CRISPR inference (CRISPRi) inducible knockdown system (52, 53) to develop a cadre of knockdown and complement strains in *C. trachomatis* serovar L2 (Ctr L2) targeting *ct226* and co-transcribed genes *ct225* and *ct224*. By characterizing FLI1 and LRRF1 localization in these strains, we demonstrate that simultaneous knockdown of *ct226, ct225*, and *ct224* prohibited localization of both FLI1 and LRRF1 to the inclusion, and only complementation of *ct226* restored their localization. Lastly, our data strongly suggest that Ct225 is not an Inc, as previously annotated in the literature, which instead localizes within the bacterial membrane during infection. Ultimately our data demonstrate that Ct226 is central for FLI1 and LRRF1 localization to the inclusion, though our results also suggest an alternative localization mechanism for FLI1 that is LRRF1-Ct226 independent, which will have implications for FLI1 function at the inclusion membrane.

## RESULTS

### FLI1 localizes to the inclusion during early mid-cycle chlamydial development

**Fig 1:**
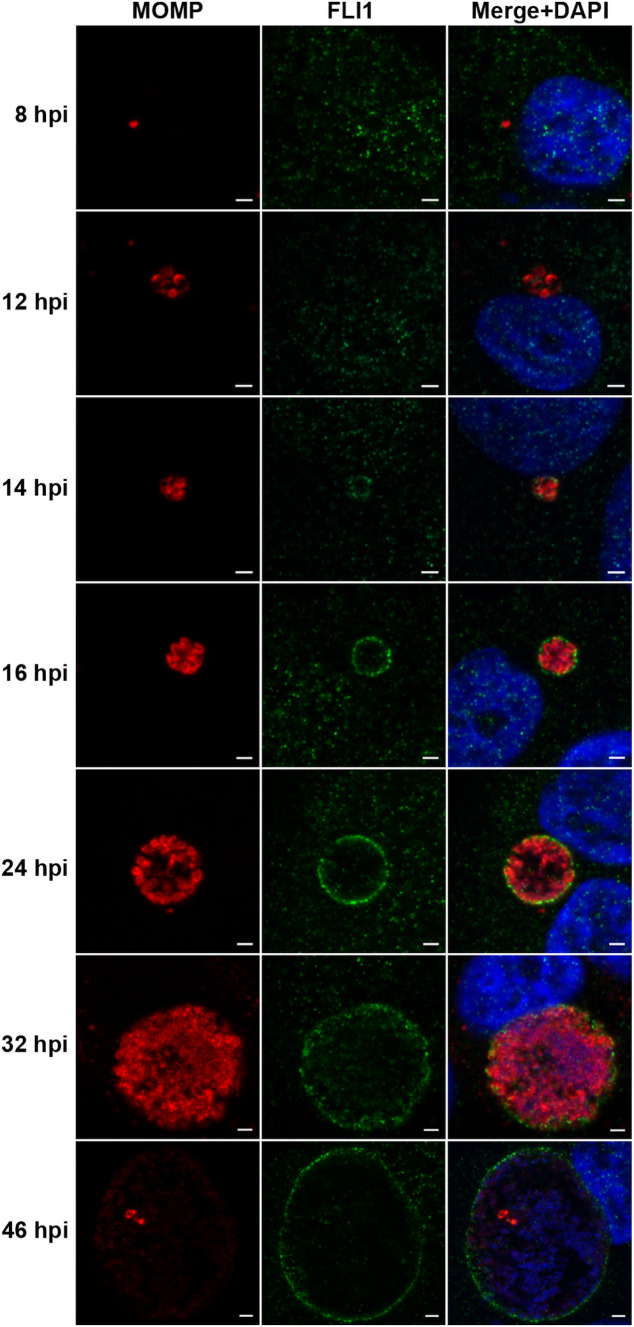
Timeline of FLI1 localization to the chlamydial inclusion. (**A**) Time-course of FLI1 recruitment to the inclusion. HEp2 cells were plated and infected with wild-type *Chlamydia trachomatis* serovar L2, as described in Materials and Methods. Coverslips were recovered and fixed with 4% paraformaldehyde at the indicated timepoints. Fixed coverslips were permeabilized with methanol and stained for indirect immunofluorescence to visualize FLI1 (anti-FLI1; green), chlamydial organisms (anti-MOMP; red), and DNA (4′,6-diamidino-2-phenylindole [DAPI]; blue). Images shown are representative of three biological replicates. Scale bar = 2 µm.

Our previous study determined that LRRF1 recruitment to the inclusion membrane begins during mid-cycle (by 12 hpi) and remains at the inclusion through late cycle timepoints (examined through 36 hpi) (28). We hypothesized that if FLI1 localization to the inclusion is dependent upon LRRF1 localization, then FLI1 localization will follow a similar temporal pattern. To determine when FLI1 localizes to the inclusion membrane, HEp2 cells were infected with wild-type Ctr L2, then fixed at timepoints between 8 and 46 hpi and processed for indirect immunofluorescence. At 14 hpi, FLI1 was apparent in small patches around the inclusion membrane and continued to localize to the inclusion throughout chlamydial development up to 46 hpi (Fig. 1). These timepoints are consistent with the temporal pattern of LRRF1 localization to the chlamydial inclusion.

### FLI1 and LRRF1 localize independently of each other yet interact together in complex with Ct226

**Fig 2:**
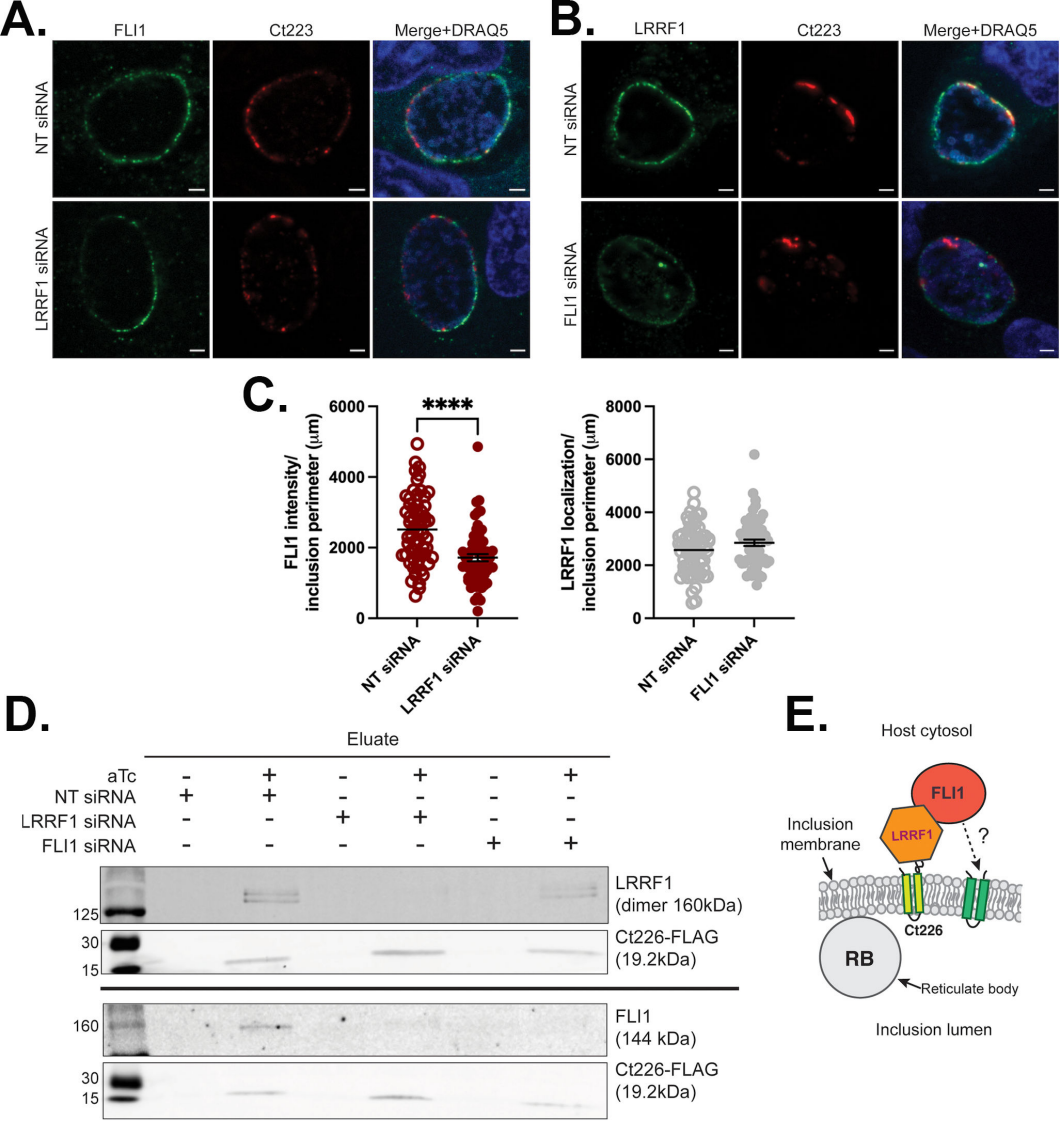
FLI1 and LRRF1 localize independently of each other to the inclusion membrane but interact in complex with Inc protein Ct226. (**A–C**) HeLa cells were treated with either non-targeting (NT), LRRF1 or FLI1 siRNA, infected with wild-type Ctr serovar L2 and fixed with 4% paraformaldehyde at 24 hpi. Indirect immunofluorescence was used to visualize (**A**) FLI1 (anti-FLI1; green) or (**B**) LRRF1 (anti-LRRF1; green), and Inc protein Ct223 (anti-Ct223; red), which is organized in microdomains around the inclusion. DRAQ5 was used to visualize host and bacterial DNA. Scale bar = 2 µm. (**C**) The intensity of FLI1 or LRRF1 localization to the inclusion after LRRF1 siRNA or FLI1 siRNA, respectively, was quantified for 60 total inclusions from two independent experiments. Results were graphed and statistically analyzed in GraphPad Prism using a Welch’s t test; ****, *P* < 0.0001. (**D**) Co-immunoprecipitation of FLI1 and LRRF1 with Ct226-FLAG. HEp2 cells were treated with either NT siRNA, FLI1 siRNA, or LRRF1 siRNA followed by infection with *C. trachomatis* serovar L2 carrying pBOMB4-Ct226-FLAG. Ct226-FLAG expression was induced with 5 nM aTc at 7 hpi and cell lysates were collected at 24 hpi. Ct226-FLAG was affinity purified and eluate fractions were blotted for FLI1 (144 kDa), LRRF1 (dimer, 160 kDa), and Ct226-FLAG (19.2 kDa). Images shown are representative of three biological replicates. (**E**) Diagram to illustrate proposed FLI1-LRRF1-Ct226 interactions at the inclusion membrane.

LRRF1 and FLI1 are known binding partners within eukaryotic cells (54–56). Previous studies demonstrated that LRRF1 interacts with chlamydial Inc protein Ct226 and that overexpression of Ct226-FLAG increases both LRRF1 and FLI1 recruitment to the inclusion (28). Therefore, we hypothesized that localization of both proteins may be dependent on each other. To test this, we performed small interfering RNA (siRNA) knockdown of either FLI1 or LRRF1, infected with wild-type Ctr L2, and used immunofluorescence to determine localization of the other protein. siRNA knockdown of FLI1 and LRRF1 was confirmed by indirect immunofluorescence (Fig. S1) and/or western blot (Fig. S2A through C). Transfection of LRRF1 siRNA or FLI1 siRNA routinely resulted in 97% (LRRF1) and 70% (FLI1) knockdown efficiency, with limited impact on the protein levels of the non-targeted binding partner (FLI1 or LRRF1, respectively) (Fig. S2C). Following siRNA knockdown of LRRF1, FLI1 localization to the inclusion membrane was still observed, although the overall intensity was significantly decreased by 32% (Fig. 2A and C). In contrast, siRNA knockdown of FLI1 did not impact LRRF1 localization to the inclusion (Fig. 2C). These data suggest that FLI1 and LRRF1 can localize to the inclusion membrane independently of each other, but that FLI1 localization to the inclusion is most efficient when LRRF1 is also present.

Next, we tested whether FLI1 can interact with Ct226 by infecting HEp2 cells with a Ctr L2 strain transformed with an inducible Ct226-FLAG construct, pBOMB4-*ct226-FLAG*. Ct226-FLAG expression was induced or not with anhydrotetracycline (aTc) at 7 hpi, and Ct226-FLAG was affinity purified from lysates collected at 24 hpi. FLI1 was detected in the eluate fraction of induced samples along with LRRF1 (Fig. S2D). These data indicate that FLI1 can interact either directly or indirectly with Ct226. To determine if LRRF1 and FLI1 can independently interact with Ct226, HEp2 cells were treated with siRNA targeting either LRRF1 or FLI1 then infected with Ctr L2 strain expressing Ct226-FLAG. Cell lysates were collected at 24 hpi, and Ct226-FLAG was affinity purified. After FLI1 siRNA knockdown, LRRF1 co-immunoprecipitated with Ct226 (Fig. 2D). However, in the absence of LRRF1, FLI1 did not co-immunoprecipitate with Ct226 (Fig. 2D), even though it is still recruited to the chlamydial inclusion in the absence of LRRF1 (Fig. 2A). Therefore, these results indicate FLI1 cannot interact with Ct226 independently of LRRF1, suggesting that there may be additional mechanisms driving FLI1 recruitment to the inclusion that are independent of direct binding to Ct226 (Fig. 2E).

### Development of knockdown and complement strains targeting *ct226* and co-transcribed genes, *ct225* and *ct224*

**Fig 3:**
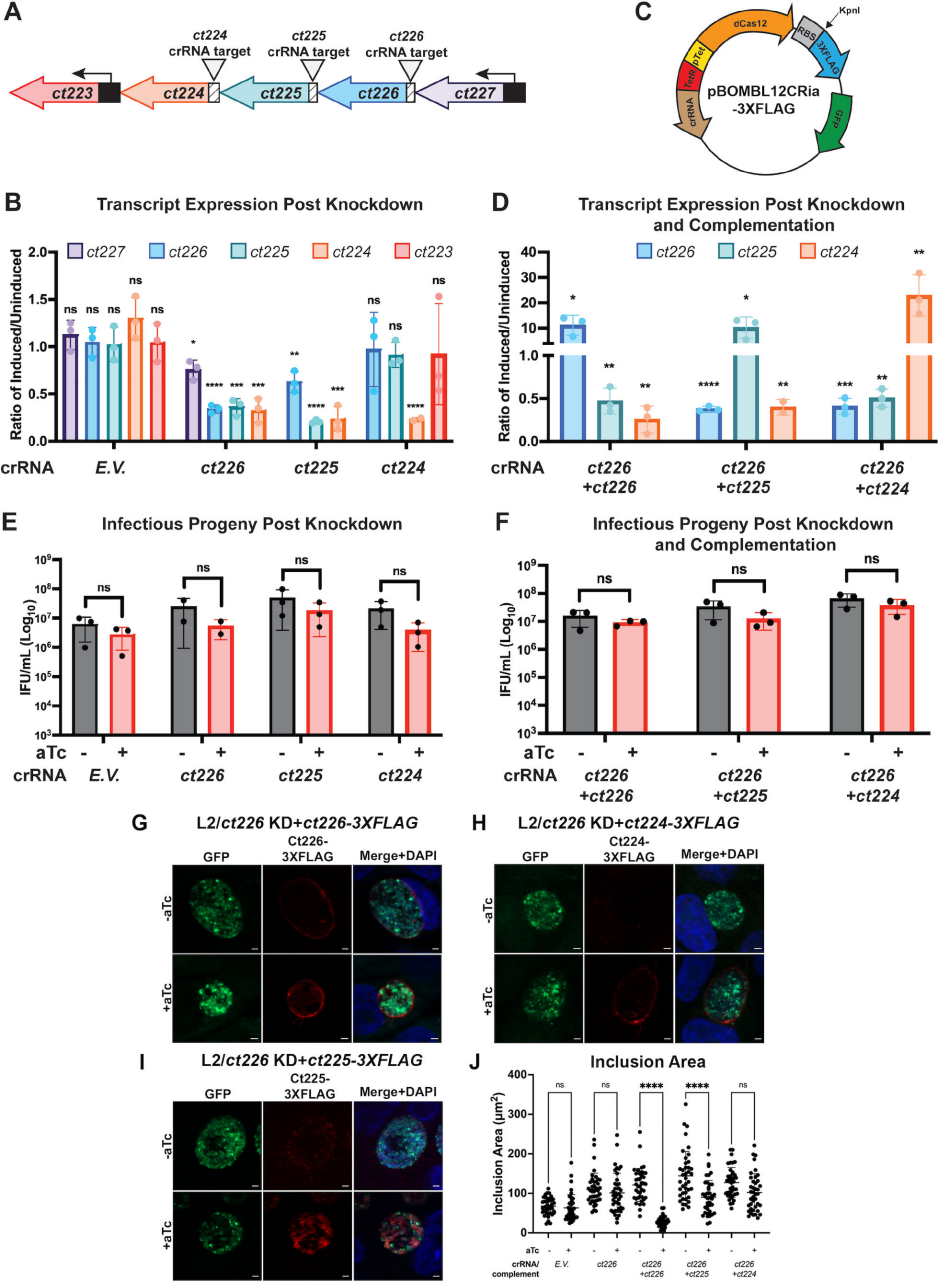
Characterization of knockdown and complementation strains by RT-qPCR, infectious progeny production, and indirect immunofluorescence microscopy. (**A**) Diagram of the *ct227* operon. Triangles indicate the binding site of the indicated crRNA in the intergenic region upstream of the targeted gene. (**B**) RT-qPCR analysis of knockdown strains. HEp2 cells were seeded, infected with the indicated strains, and induced as described in Materials and Methods. RNA was collected and processed for RT-qPCR. cDNA transcript levels of genes were normalized to 16s transcripts and reported as the ratio of induced to uninduced at 12 hpi. A paired Student’s *t*-test was used to determine statistically significant differences in transcript levels between uninduced and induced conditions for each strain. (**C**) Diagram of pBOMBL12CRia-3XFLAG vector design for complementation downstream of dCas12. (**D**) RT-qPCR analysis of complement strains. Samples were collected and analyzed as described above. (**E**) Infectious progeny production at 24 hpi in knockdown strains. Infectious progeny were measured in both uninduced and induced inclusions and reported as IFU per milliliter and a paired Student’s *t*-test was used to determine statistical significance between uninduced and induced conditions for each strain. (**F**) Infectious progeny production in complementation strains. (**G–I**) Indirect immunofluorescence confirming expression and localization of the complemented 3×FLAG-tagged Inc for all three complement strains. HEp2 cells were seeded on glass coverslips, infected with appropriate complement strain, and induced as described in the Materials and Methods. At 24 hpi, the coverslips fixed for indirect immunofluorescence to visualize the 3×FLAG-tagged Inc (anti-FLAG; red) and host and chlamydial DNA (4′,6-diamidino-2-phenylindole [DAPI]; blue). Constitutively expressed GFP was used to visualize the chlamydial organisms (GFP; green). (**J**) Quantification of inclusion area in complementation strains by ImageJ. An ordinary two-way analysis of variance test was performed to identify statistically significant differences in inclusion area. Scale bar = 2 mm. For graphs, statistical significance was reported as follows: *P < 0.05, **P < 0.01, ***P < 0.001, ****P < 0.0001; ns, not significant. Immunofluorescence images shown are representative of three biological replicates. For infectious progeny and transcripts, data from three biological replicates are plotted.

To test the hypothesis that knockdown of *ct226* would inhibit recruitment of LRRF1, but not FLI1, to the chlamydial inclusion, we created an inducible knockdown strain targeting *ct226* using a dCas12-based CRISPRi inducible knockdown system (53). The principle of CRISPRi relies on a catalytically dead Cas12 enzyme, which is guided to the target genetic sequence by a CRISPR RNA (crRNA) (52, 53) to sterically inhibit transcription at that site. Into the pBOMBL12CRia vector, we cloned a crRNA targeting the intergenic region upstream of *ct226* to create the vector pBOMBL12CRia(*ct226*) (Fig. 3A), and transformed the resulting plasmid into Ctr L2, herein referred to as the L2/*ct226* KD strain. We also included the Ctr L2 strain transformed with the empty vector plasmid, pBOMBL12CRia (*empty vector* [*E.V*.]), as the negative control. The L2/*E.V*. strain maintains inducible expression of dCas12 but lacks a targeting crRNA. We validated knockdown using quantitative reverse transcription-polymerase chain reaction (RT-qPCR) to measure transcript levels and performed inclusion forming unit (IFU) assays to determine if chlamydial development was grossly affected.

To measure transcript levels and validate knockdown, HEp2 cells were infected with either L2/*ct226* KD or L2/*E.V*. strain, and knockdown was induced using aTc as described in Materials and Methods. RNA was collected at 3 hpi (time of aTc addition), 12 hpi (peak transcription of *ct226*), and 24 hpi (*ct226* transcription returns to baseline). These timepoints also capture transcription at early, mid, and late cycle of chlamydial development, respectively (15). *ct226* is located in the *ct227* operon, where genes *ct227, ct226, ct225,* and c*t224* are co-transcribed (Fig. 3A). Therefore, genes upstream and downstream of *ct226* were also measured to determine any polar effects of knockdown. Consistent with its use as a negative control strain, induction of the L2/*E.V*. strain with aTc did not result in differences in transcript levels *of ct227, ct226, ct225*, or *ct224* (Fig. 3B). Transcripts of *ct223,* located downstream of the *ct227* operon, were also unaffected (Fig. 3B). Upon induction of knockdown in the L2/*ct226* KD strain, RT-qPCR analysis demonstrated ~66% knockdown of *ct226* transcripts at 12 hpi when compared with the uninduced samples (Fig. 3B). Transcripts for downstream genes *ct225* and *ct224* also showed 64% and 67% knockdown, respectively, and transcripts of upstream gene *ct227* demonstrated a 24% decrease compared to the uninduced levels (Fig. 3B). Therefore, targeting *ct226* by CRISPRi significantly represses transcription of *ct226*, *ct225*, and *ct224*.

Given the polar effects on *ct225* and *ct224*, we created and characterized two additional knockdown strains that were transformed with plasmids carrying crRNA that specifically targeted these respective genes (Fig. 3A), herein referred to as L2/*ct225* KD and L2/*ct224* KD strains, respectively. Following induction of the L2/*ct225* KD strain, at 12 hpi, both *ct225* (~79%) and *ct224* (~76%) transcripts were decreased, and a 37% decrease in *ct226* transcripts was observed (Fig. 3B). Induction of the L2/*ct224* KD strain resulted in a 77% decrease in *ct224* transcripts while *ct226* and *ct225* transcript levels were unchanged. The transcript levels of the immediate downstream gene, *ct223*, were also unchanged in both strains (Fig. 3B).

To assess the contribution of each individual Inc in the absence of the others, we individually complemented *ct226, ct225,* or *ct224* in the L2/*ct226* KD background. For complementation, we modified the pBOMBL12CRia(*ct226*) vector by inserting a KpnI digest site and 3×FLAG tag directly 3´ of dCas12 (Fig. 3C). The KpnI site allowed complementation of each individual Inc under the same inducible promoter as dCas12. The three resulting vectors were transformed into Ctr L2 to create the three complement strains. By RT-qPCR analysis, induction of dCas12 and complementation of Ct226-3×FLAG in the L2/*ct226* KD+*ct226-3×FLAG* strain led to an ~8.7-fold increase in *ct226* transcripts and a 62.9% and 81.6% decrease in *ct225* and *ct224* transcripts, respectively, compared to the uninduced samples (Fig. 3D). For complementation of Ct225-3×FLAG, treatment with aTc in the L2/*ct226* KD+*ct225-3×FLAG* strain led to a 10-fold increase in *ct225* transcripts compared to uninduced conditions, whereas both *ct226* (62% decrease) and *ct224* (60% decrease) transcript levels decreased following induction (Fig. 3D). Finally, for complementation of Ct224-3×FLAG, treatment with aTc in the L2/*ct226* KD+*ct224-3×FLAG* strain led to ~23-fold increase in *ct224* transcripts, while *ct226* and *ct225* transcripts were decreased 59% and 49%, respectively (Fig. 3D).

To determine if CRISPRi targeting of these genes would grossly impact chlamydial development, we performed IFU assays (Fig. 3E and F). Notably, induction of knockdown in L2/*E.V*. strain demonstrated a ~48% decrease in infectious progeny (uninduced 6.08 × 10^6^ IFU/mL; induced 2.79 × 10^6^ IFU/mL; Fig. 3E), consistent with increased energy requirements likely associated with dCas12 expression (57). For the L2/*ct226* KD strain, induction of knockdown reduced the yield of infectious progeny by 69% (uninduced 1.87 × 10^7^ IFU/mL; induced 4.14 × 10^6^ IFU/mL; Fig. 3E). In comparison to the negative control, induction of knockdown in the L2/*ct226* KD strain resulted in only a 21% decrease and is not considered biologically significant (i.e., 1-log reduction in IFUs). Induction of the L2/*ct225* KD strain resulted in a 60% reduction in IFUs (uninduced 4.82 × 10^7^ IFU/mL; induced 1.77 × 10^7^ IFU/mL) when compared to uninduced conditions and a 12% decrease compared to the L2/*E.V* strain (Fig. 3E). Knockdown of *ct224* resulted in an 80% decrease in IFUs compared to uninduced conditions (uninduced 2.04 × 10^7^ IFU/mL; induced 3.80 × 10^6^ IFU/mL) and 32% decrease compared to the negative control (Fig. 3E), which is the greatest reduction across all three knockdown strains but is still not considered biologically relevant. Induction of knockdown and complementation in the L2/*ct226* KD+*ct226-3×FLAG* strain resulted in a 48.6% decrease in the number of infectious progeny (uninduced 1.54 × 10^7^ IFU/mL; induced 8.97 × 10^6^ IFU/mL; Fig. 3F). For the L2/*ct226* KD*+ct225-3×FLAG* strain, recovered infectious progeny demonstrated a 46% decrease following treatment with aTc (uninduced 3.28 × 10^7^ IFU/mL; induced 1.20 × 10^7^ IFU/mL; Fig. 3F). Inducing expression of Ct224-3×FLAG in the L2/*ct226* KD*+ct224-3×FLAG* strain yielded a 58% decrease in infectious progeny compared to uninduced conditions (4.81 × 10^7^ IFU/mL; induced 2.74 × 10^7^ IFU/mL; Fig. 3F). Overall, these data suggest that knockdown of any or all of *ct226, ct225*, or *ct224* has a minimal impact on developmental cycle progression in the tested experimental conditions.

Lastly, we used indirect immunofluorescence to assess expression and localization of the complemented Inc proteins. In uninduced conditions, the L2/*ct226* KD+*ct226-3×FLAG* strain demonstrated some leaky expression of Ct226-3×FLAG both by immunofluorescence and by western blot (Fig. 3G; Fig. S3). Following induction, Ct226-3×FLAG is expressed and secreted, resulting in localization to the inclusion membrane (Fig. 3G). Similar results were obtained after induction of Ct224-3×FLAG, with the protein localizing to the inclusion membrane, where it appeared concentrated within microdomains (a pattern of localization where Incs localize in discrete clusters within the inclusion membrane [58]) (Fig. 3H). Surprisingly, induction of the L2/*ct226* KD+*ct225-3×FLAG* strain with aTc resulted in Ct225-3×FLAG expression, but the construct localized to the bacterial membrane and not the inclusion membrane (Fig. 3I). Ct225 is annotated in the literature as an Inc protein, so this was an unexpected finding (59, 60). To ensure that overexpression of Ct225 in the absence of Ct226 or Ct224 was not preventing type III secretion of Ct225, wild-type Ctr L2 was transformed with a plasmid encoding Ct225 fused to a 3×FLAG tag (Ctr L2 Ct225-FLAG). Induction of exogenous expression of Ct225-FLAG in a wild-type background also resulted in Ct225-FLAG localizing to the chlamydial membrane, not the inclusion membrane (Fig. S4A and B). Examination of endogenous Ct225 localization by immunofluorescence in both wild-type Ctr L2 and induced Ctr L2 Ct225-FLAG strains demonstrated that endogenous Ct225 appears within chlamydial bacteria (Fig. S4C) and colocalizes with exogenously expressed Ct225-FLAG (Fig. S4D). Taken together, these data suggest that Ct225 is not an Inc protein as previously annotated.

During the initial characterization of our single complement strains, we consistently observed that induction of the L2/*ct226* KD+*ct226-3×FLAG* strain resulted in smaller inclusions (Fig. 3G and J). Therefore, we quantified inclusion area to determine if complementation of individual Incs into the L2/*ct226* KD strain background altered inclusion size. Neither L2/*E.V*. nor L2/*ct226* KD strains demonstrated a difference in inclusion size between uninduced and induced conditions (Fig. 3J). Therefore, neither dCas12 expression nor simultaneous knockdown of *ct226, ct225,* and *ct224* altered inclusion growth. However, single complementation of Ct226-3×FLAG, in the absence of Ct225 and Ct224, decreased inclusion area by 77% (Fig. 3J). Complementation of Ct225-3×FLAG also had a moderate impact on inclusion area (37% decrease), while complementation of Ct224-3×FLAG had no impact on inclusion area (Fig. 3J). These data demonstrate that expression of Ct226 in the absence of Ct225 and Ct224 significantly reduces inclusion size.

### Induction of knockdown in L2/*ct226* KD and L2/*ct225* KD, but not L2/*ct224* KD, negatively impacts FLI1 and LRRF1 localization to the inclusion membrane

**Fig 4:**
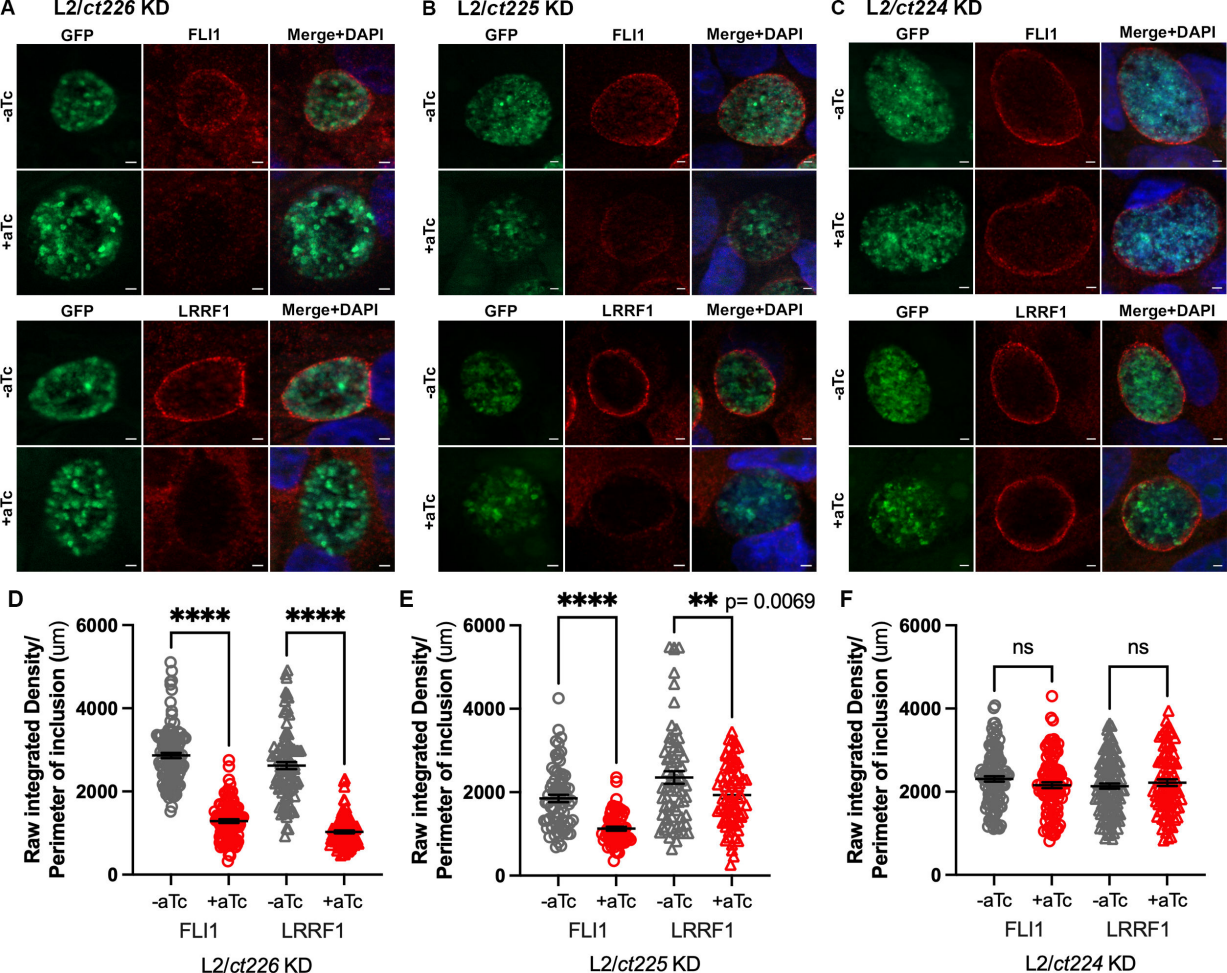
Knockdown of *ct226* and *ct225*, but not *ct224*, negatively impacts localization of FLI1 and LRRF1 to the chlamydial inclusion membrane. (**A–C**), Representative images of localization of FLI1 and LRRF1 in knockdown strains (**A**) L2/*ct226* KD, (**B**) L2/*ct225* KD, and (**C**) L2/*ct224* KD at 24 hpi. Coverslips were imaged at 100× to visualize chlamydial organisms (GFP; green), FLI1 (anti-FLI1; red) or LRRF1 (anti-LRRF1; red), and host and bacterial DNA (DAPI; blue). Scale bar = 2 mm. (**D–F**) ImageJ/Fiji quantification of fluorescence intensities for FLI1 and LRRF1 at the inclusion membrane. Raw integrated density of either FLI1 or LRRF1 was normalized to inclusion perimeter measurements and the mean and standard error of the mean from three independent experiments, representing greater than 65 inclusions total were graphed using GraphPad Prism. Values were statistically analyzed using an ordinary one-way analysis of variance with Šidák’s multiple comparisons test. ****P < 0.0001; **P = 0.0069.

To test the hypothesis that *ct226* knockdown inhibits LRRF1, but not FLI1, localization to the inclusion, localization of FLI1 and LRRF1 to inclusions formed by L2/*ct226* KD, L2/*ct225* KD, and L2/*ct224* KD after induction of knockdown by aTc was determined by indirect immunofluorescence. HEp2 cells were infected, knockdown was induced or not at 3 hpi, and cells were fixed at 24 hpi and processed for immunofluorescence. aTc treatment of HEp2 cells infected with the L2/*E.V*. strain did not inhibit localization of FLI1 or LRRF1 to the inclusion membrane (Fig. S5). Of note, FLI1 and LRRF1 localization to the inclusion membrane is observed for inclusions from all knockdown strains in the absence of aTc (Fig. 4A through C, top row). However, when knockdown of *ct226* was induced, FLI1 and LRRF1 remained in the host cell cytosol and did not localize to the inclusion membrane of the L2/*ct226* KD strain (Fig. 4A). Quantification of these images demonstrated a statistically significant decrease of FLI1 and LRRF1 localization to the chlamydial inclusion during knockdown of *ct226* compared to wild-type conditions (Fig. 4D; ****P < 0.0001). Interestingly, induction of the L2/*ct225* KD strain with aTc resulted in a statistically significant decrease in fluorescence intensity of both FLI1 (****P < 0.0001) and LRRF1 (**P = 0.0069) at the inclusion membrane (Fig. 4B and E). Average fluorescence intensity of FLI1 decreased by 45% under induced conditions compared to uninduced conditions, while the fluorescence intensity of LRRF1 decreased only by 20% (Fig. 4E). Lastly, knockdown of Ct224 did not diminish localization of FLI1 or LRRF1 localization to the inclusion (Fig. 4C and F). In summary, these data suggest that simultaneous knockdown of *ct226* and *ct225* (as in the induced L2/*ct226* KD strain) results in total loss of FLI1 and LRRF1 proteins at the inclusion. These results were unexpected given that we have demonstrated that Ct225 does not localize to the chlamydial inclusion membrane; and thus, it is unclear how the presence or absence of Ct225 would impact recruitment of host proteins to the inclusion.

### Complementation of Ct226-3×FLAG rescues localization of both FLI1 and LRRF1

**Fig 5:**
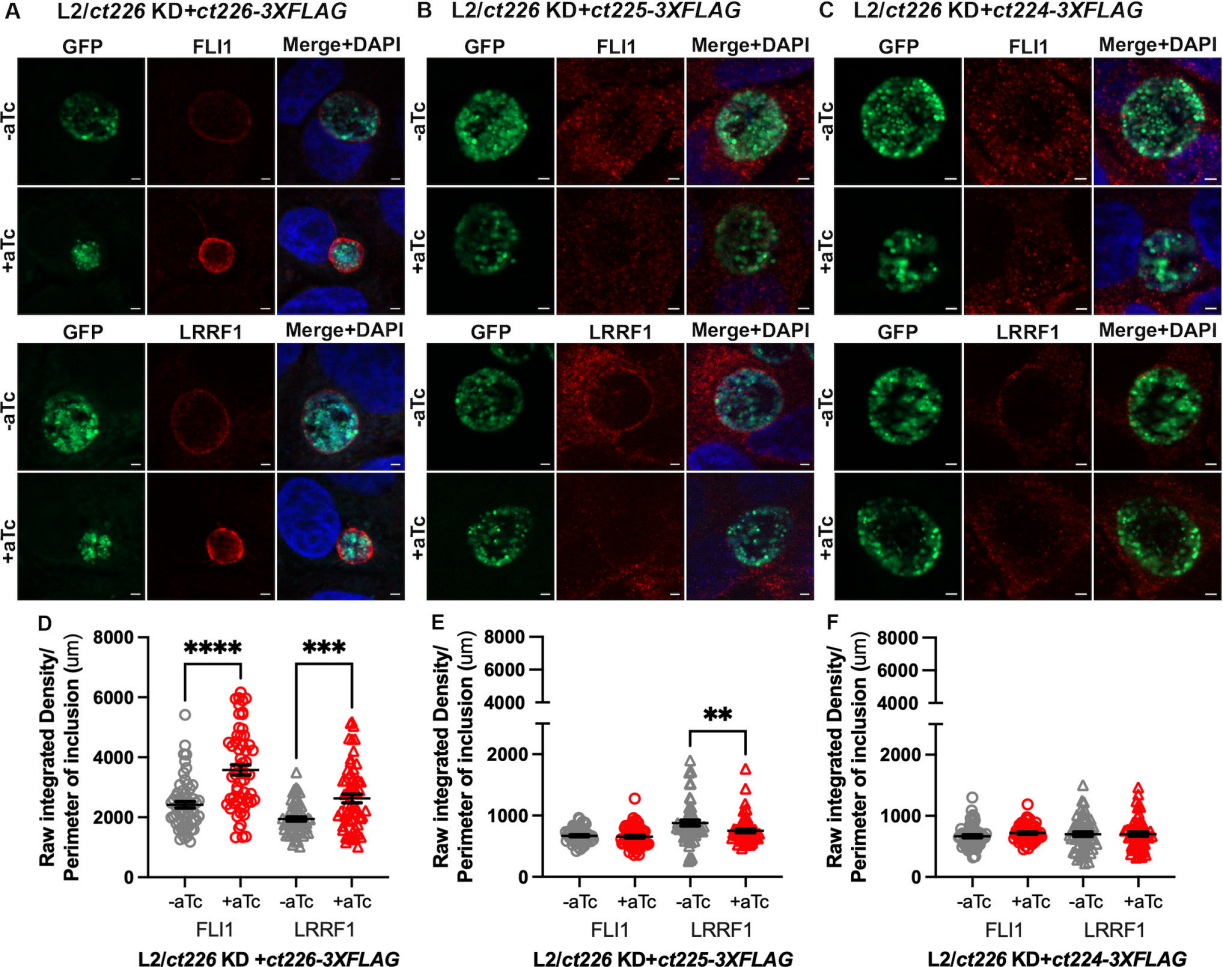
Complementation of Ct226, but not Ct225 nor Ct224, fully restores both LRRF1 and FLI1 localization to the chlamydial inclusion. (**A–C**). Representative images of localization of FLI1 and LRRF1 in complementation strains (**A**) L2/*ct226* KD+*ct226-3×FLAG,* (**B**) L2/*ct226* KD+*ct225-3×FLAG*, and (**C**) L2/*ct226* KD+*ct224-3×FLAG* at 24 hpi. Coverslips were imaged at 100× to visualize the chlamydial organisms (GFP; green), FLI1 (anti-FLI1; red) or LRRF1 (anti-LRRF1; red), and host and bacterial DNA (DAPI; blue). Scale bar = 2 mm (**D–F**), ImageJ/Fiji quantification of fluorescence intensities for FLI1 and LRRF1 at the inclusion membrane where raw integrated density normalized to inclusion perimeter measurements with mean and SEM from three independent experiments, representing 60 inclusions total, were graphed using GraphPad Prism. Values were statistically analyzed using an ordinary one-way analysis of variance with Šidák’s multiple comparisons test. In panel D, ****P < 0.0001; ***P = 0.0004. In panel E, **P = 0.0067.

Based on our above results, we hypothesized that complementation of Ct226 in the L2/*ct226* KD strain would fully restore FLI1 and LRRF1 localization to the inclusion. Furthermore, we postulated that complementation of Ct225 would partially restore FLI1 and LRRF1 recruitment to the inclusion. Hence, we used the individual complement strains described above to infect HEp2 cells, which were then fixed at 24 hpi and processed for indirect immunofluorescence as described above. In uninduced cultures, monolayers infected with the L2/*ct226* KD+*ct226-3×FLAG* strain demonstrated both FLI1 and LRRF1 localized at the inclusion membrane, consistent with wild-type expression of Ct226 and downstream gene products (first and third rows, Fig. 5A). Addition of aTc, and simultaneous induction of *ct226*/*ct225*/*ct224* KD and expression of Ct226-3×FLAG resulted in FLI1 and LRRF1 localization to the inclusion (Fig. 5A). The overall signal of FLI1 and LRRF1 appeared less intense in the controls due to all images being taken at the same exposure and the signals for both FLI1 and LRRF1 being more intense during induction of the L2/*ct226* KD+*ct226-3×FLAG* strain. Quantification of these images demonstrated that individual complementation of Ct226 results in localization of FLI1 and LRRF1 at the inclusion (Fig. 5D). Complementation of the L2/*ct226* KD strain with either CT225-3×FLAG or CT224-3×FLAG yielded interesting results. In the absence of aTc, FLI1 did not localize at all to inclusions formed by the L2/*ct226* KD+*ct225-3×FLAG* or the L2/*ct226* KD+*ct224-3×FLAG* strains (top rows, Fig. 5B and C). These results were reproducible and quantifiable (Fig. 5E and F, -aTc). These results were also unexpected because FLI1 localizes to inclusions formed by the parent strain (L2/*ct226* KD) in uninduced conditions (Fig. 4A and Fig. 5A, top row). Also, LRRF1 localizes as predicted to inclusions formed by L2/*ct226* KD+*ct225-3×FLAG* but does not reliably localize to inclusions formed by L2/*ct226* KD+*ct224-3×FLAG* strains in the absence of aTc (Fig. 5B and C). The only alterations to the original pBOMBL12CRia(*ct226*) plasmid were an additional ribosomal binding site, KpnI restriction site (for insertion of *ct226, ct225,* or *ct224*), and a 3×FLAG epitope (Fig. 3C). We cannot rule out that the cause of these unusual phenotypes may be the result of the epitope tag sequence or the combination of *ct225-3×FLAG* or *ct224-3×FLAG* sequences. Induction of complementation of Ct225-3×FLAG or Ct224-3×FLAG from the L2/*ct226* KD strain resulted in FLI1 and LRRF1 remaining in the host cytosol and not localizing to the chlamydial inclusion (Fig. 5B and C). The addition of aTc, in and of itself, does not alter FLI1 or LRRF1 recruitment to the inclusions formed by wild-type L2 (Fig. S5). As expected, single complementation of Ct225 does not rescue LRRF1 localization to the inclusion during Ct226 knockdown (Fig. 5B and E). Unfortunately, given the behavior of the controls in this experiment, no clear conclusions can be made about the ability of Ct225 or Ct224 single complementation to influence the recruitment of FLI1 to the inclusion or Ct224 to influence the recruitment of LRRF1.

### In the absence of LRRF1, complementation of Ct226-3×FLAG rescues FLI1 localization to the inclusion membrane

**Fig 6:**
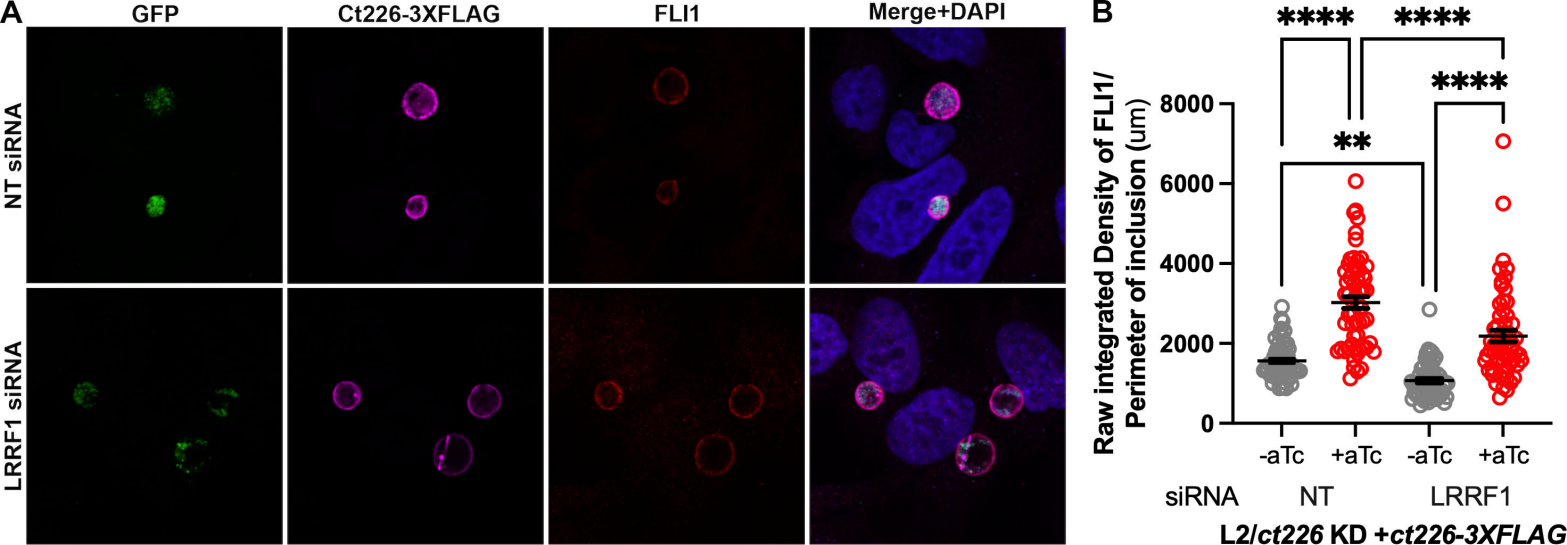
In the absence of LRRF1, complementation of Ct226-3×FLAG into the L2/*ct226* KD strain rescues FLI1 localization to the inclusion membrane. (**A**) Representative images from two independent experiments of HEp2 cells treated with either NT or LRRF1 siRNA and then infected with the L2/*ct226* KD+*ct226-3×FLAG* strain, which was induced for dCas12 and Ct226-3×FLAG expression. Twenty-four hours post infection, samples were imaged at 60× magnification to visualize chlamydial organisms (GFP; green), Ct226-3×FLAG (anti-FLAG; magenta), FLI1 (anti-FLI1; red), and host and bacterial DNA (DAPI, blue). (**B**). ImageJ/Fiji quantification of fluorescence intensities from 60 inclusions for FLI1 at the inclusion membrane where raw integrated density normalized to inclusion perimeter measurements with mean and SEM were graphed with GraphPad Prism. Values were statistically analyzed using an ordinary one-way analysis of variance with Šidák’s multiple comparisons test. ****P < 0.0001; **P = 0.0068.

To definitively test if FLI1 localizes to the inclusion independently of LRRF1, we transfected HEp2 cells with non-targeting (NT) or LRRF1 siRNA, then infected with the L2/*ct226* KD+*ct226-3×FLAG* strain. At 3 hpi, cells were treated or not with aTc then, at 24 hpi, were either fixed for immunofluorescence microscopy or lysed and prepared for western blot analysis. LRRF1 knockdown was confirmed by both indirect immunofluorescence and western blot (Fig. S6A and B). Consistent with our previous results*,* FLI1 localized to the inclusion membrane under Ct226 complementation conditions in cells treated with NT siRNA (Fig. 6A). FLI1 also localized to inclusions during Ct226 complementation in the absence of LRRF1 that had been depleted by siRNA treatment (Fig. 6A). Quantification of these images revealed that, in general, localization of FLI1 to chlamydial inclusions was less during LRRF1 knockdown (-aTc, NT vs LRRF1 siRNA, *P* = 0.0067, Fig. 6B). However, upon induction of *ct226* knockdown and simultaneous complementation of Ct226-3×FLAG, FLI1 intensity levels at the inclusion significantly increased (Fig. 6B). These data confirm that FLI1 has an additional, possibly redundant, mechanism of recruitment to the inclusion membrane that is distinct from LRRF1-mediated recruitment. These findings have implications for how FLI1 may be functioning at the inclusion and highlight a possible reason as to the difficulty in identifying a functional phenotype of FLI1 or LRRF1 during chlamydial infection.

## DISCUSSION

Of all chlamydial effector proteins, candidate *inc* genes make up an estimated 7% of open reading frames within the highly reduced chlamydial genome (61), highlighting their importance to chlamydial pathogenesis. Building on our previous studies (28), we sought to describe the Inc-host protein interactions required for recruitment of FLI1 and LRRF1—two eukaryotic proteins that are involved in multiple cell-signaling networks and regulatory processes. We hypothesize that their stable localization at the inclusion means these proteins are either (i) being sequestered away from their native functions, or (ii) their functions are being relocated to the inclusion membrane. Understanding how these proteins localize to the inclusion is an important first step toward understanding their function.

In this work, we report the dynamics of FLI1 and LRRF1 localization to the inclusion membrane and identify the chlamydial proteins involved in their recruitment. We demonstrated FLI1 localizes to the inclusion membrane at 14 hpi, (early mid-cycle of chlamydial development), and it remains at the inclusion throughout chlamydial development, which correlates with the temporal pattern of LRRF1 localization to the inclusion (28). Furthermore, we demonstrated FLI1 interacts with Ct226 in complex with LRRF1, and while FLI1 depends on LRRF1 for interaction with Ct226, both proteins can localize to the inclusion independently of each other. Together, these data suggest multiple mechanisms for FLI1 localization aside from interaction with Ct226 and LRRF1.

Of note, two other ongoing studies in the field have implicated Ct226 in FLI1 recruitment to the inclusion in an LRRF1-dependent manner. Interestingly, these studies from the Lutter group and Engel group use different genetic methods to knockout (KO) *ct226* in contrast to our inducible CRISPRi knockdown approach. For example, the Lutter group has generated a *ct226* knockout strain using type II intron mutagenesis (i.e., TargeTron [62]) (E. Lutter, C. Holcomb, personal communication), while the Engel team has created a *ct226* knockout strain using fluorescence-reported allelic exchange mutagenesis (63) (J. Engel, C. Elwell, personal communication). Indeed, some of our results agree with their findings. For example, we demonstrated that simultaneous knockdown of *ct226*, *ct225*, and *ct224* resulted in a complete loss of localization of either FLI1 or LRRF1 during chlamydial infection and only individual complementation of Ct226 in this background fully restored FLI1 and LRRF1 localization. These data would suggest that expression of Ct226 alone leads to FLI1 localization to the chlamydial inclusion. However, we were unable to detect a direct interaction between Ct226 and FLI1 via co-immunoprecipitation. FLI1 was only found in a Ct226-FLAG eluate if LRRF1 was present (Fig. 2D). In addition, we observed that FLI1 can localize to Ct226-positive (i.e., wild-type) inclusions in the absence of LRRF1, but the intensity of FLI1 is significantly decreased by 32% (Fig. 2A and C). Similarly, expression of Ct226-3×FLAG following knockdown of *ct226* in LRRF1 siRNA-treated cells resulted in recruitment of FLI1 to the inclusion (Fig. 6). Taken together, these data are strong evidence for an additional, LRRF1-independent recruitment mechanism for FLI1 that does not rely on a direct protein-protein interaction with Ct226. These data also suggest that Ct226 interacts with another eukaryotic protein or Inc that recruits FLI1 to the inclusion membrane.

It is intriguing that simultaneous knockdown of three genes (*ct226, ct225,* and *ct224*) in the L2/*ct226* KD strain did not alter inclusion area, but individual complementation of Ct226-3×FLAG expression with 2 nM aTc in this genetic background produced inclusions that were 77% smaller in area compared to uninduced conditions (Fig. 3J). A previous study demonstrated that overexpression of Ct226-FLAG with 5 nM aTc in Ctr L2 led to only a 25% decrease in inclusion area (28). These data indicate that restriction of inclusion growth due to Ct226 overexpression is limited when there are endogenous levels of Ct225 and Ct224 present. It is possible that Ct226 cooperates with Ct225 and/or Ct224 for optimal inclusion growth. Another possibility is that normal expression of the entire *ct227* operon controls the final concentration of Ct226 in the inclusion membrane. Thus, overexpression of Ct226 in the absence of the other proteins may concentrate other components of the inclusion membrane and/or disrupt canonical Inc-host and Inc-Inc interactions. To test these hypotheses, combining genetic knockout with multiplexed complementation of two or more genes would help determine which Incs functionally cooperate with each other during chlamydial development. Additionally, co-infection models using strains expressing Incs tagged with different epitopes could also be used to determine interactions between Ct226 and other Incs (64, 65).

The localization of Ct225 to the chlamydial membrane, and not the inclusion membrane, was an unexpected finding in this study. Ct225 is the smallest protein encoded by the *ct227* operon (~13–15 kDa) (61, 66). All Inc proteins have a unique and distinctive structure, which feature bilobed transmembrane domains, and Ct225 was identified as a putative Inc based on this characteristic domain in the first bioinformatic screen used to identify chlamydial type III secretion effectors (20). Consistent with this study, our own analysis of the Ct225 protein sequence via UniProt clustal analysis confirmed two predicted transmembrane domains (Fig. S4E). Inclusion membrane localization was first mentioned in a study measuring temporal expression of chlamydial genes, saying Ct225 had been “detected in association with the inclusion membrane by immunofluorescent staining,” but the data were not shown (16). In 2008, Li and colleagues characterized 50 putative inclusion membrane proteins by raising antibodies against chlamydial proteins fused to glutathione S-transferase (GST) (59). Indirect immunofluorescence studies using the anti-GST-Ct225 antibody (also used in Fig. S4 of this study) demonstrated that it reacted with a protein expressed on the inclusion membrane (59). The original antibody was raised against a purified Ct225 cloned from *C. trachomatis* serovar D; however, an alignment of the amino acid sequence from serovar D and serovar L2 are identical, including the positions of the transmembrane domains (Fig. S4E). The most tangible difference between our study and previous is the fixation conditions. The original study fixed infected monolayers in 2% paraformaldehyde and permeabilized with saponin (59); in the current study, infected monolayers were fixed in either methanol or paraformaldehyde with similar results (Fig. 3I; Fig. S4A through D and F) . A previous attempt to exogenously express Ct225-FLAG from *C. trachomatis* to determine subcellular localization was not successful and unfortunately cannot be used for comparison (60).

Because our data demonstrate that Ct225 does not localize to the inclusion membrane, it is interesting that our studies showed induction of knockdown in the L2/*ct225* KD strain led to a statistically significant decrease in FLI1 and LRRF1 recruitment to the inclusion membrane (Fig. 4B and E). Since Ct225 is situated in the bacterial membrane and can interact with Ct226 by bacterial adenylate cyclase two-hybrid assay (BACTH) (Fig. S7), Ct225 may be important for efficient type III secretion of Ct226. Hence, knockdown of *ct225* might lead to decreased Ct226 in the inclusion membrane and subsequently decreased recruitment of host proteins LRRF1 and FLI1. An additional possibility is that the modest reduction of *ct226* transcription after induction of the L2/*ct225* KD strain (Fig. 3B) lowers protein levels of Ct226 enough to decrease recruitment of LRRF1 or FLI1 to the inclusion. However, studies using the strain L2/*ct226* KD+*ct226-3×FLAG* suggest that Ct225 is not required for Ct226 secretion, since Ct226-3×FLAG is secreted and localized to the inclusion membrane while Ct225 expression is diminished (Fig. 3G). As such, it is possible that Ct225 serves a positive regulatory function for Ct226 secretion, or, alternatively, it may be involved in organization and stability of Ct226 in the inclusion membrane. Future studies are needed to clarify the function and molecular interactions of Ct225 during *C. trachomatis* infection.

The goal of this study was to better understand the mechanisms of FLI1 and LRRF1 recruitment to the inclusion by Inc proteins. We established that FLI1 recruitment occurs during early mid-cycle development and interacts in complex alongside Ct226 and LRRF1. We clearly demonstrate that FLI1 relies on LRRF1 for interaction with Ct226, yet does not rely on LRRF1 for localization to the inclusion. The fact that they localize independently of each other not only has implications for their mechanism at the inclusion membrane, but also suggests that FLI1 interacts with another protein (eukaryotic or chlamydial) at this site. Both observations are critical for identifying which host signaling pathways are impacted by FLI1/LRRF1 localization and for determining how *C. trachomatis* might be modifying them to create an optimal intracellular niche. To this end, the multitude of strains developed in this study will be used to test the impact of FLI1/LRRF1 localization on host cell processes. Importantly, we have shown the adaptability of the CRISPRi knockdown system to investigate Inc-host, as well as Inc-Inc, interactions. Understanding these complex interactions and how they change throughout development will help us to understand how *C. trachomatis* manipulates its environment to establish a successful infection.

## MATERIALS AND METHODS

### Tissue culture and chlamydial strains

HEp2 cells (Ouellette lab stock), HeLa cells (CCL-2.1; American Type Culture Collection [ATCC], Manassas, VA), and McCoy cells (CRL-1696; ATCC, Manassas, VA) were routinely passaged and were cultured in Dulbecco’s modified Eagle media (DMEM; Gibco/Thermo Fisher) supplemented with 10% fetal bovine serum (Sigma-Aldrich, St. Louis, MO) and 10 µg/mL gentamicin (Gibco/Thermo Fisher). All cells were incubated at 37°C at 5% CO_2_. *Chlamydia trachomatis* serovar L2 (lymphogranuloma venereum [LGV] strain L2/434/Bu) was propagated using HeLa cells and purified for use in experiments using density gradient centrifugation as described in previous protocols (67). HeLa or McCoy cells were used for LGV 434 transformation of the strains produced in this study. Chlamydial strain titers were determined by measuring by number of IFUs, using previously described methods (68). HeLa cells were used to determine titers and titers were used to determine multiplicity of infection (MOI) for subsequent experiments. For all strains, cells were infected at the indicated MOI by centrifugation at 400 × *g* for 15 min and medium was replaced following 15-min incubation at 37°C at 5% CO_2_. All cell lines and media were routinely tested for *Mycoplasma* spp. contamination (Lookout Mycoplasma PCR detection kit; Sigma-Aldrich, St. Louis, MO).

### Nomenclature used in this study

*C. trachomatis* serovar D was the first serovar to be sequenced and annotated (61); thus, it is a common practice in the field to use serovar D nomenclature due to the sequence similarity between serovar D and serovar L2 (66). However, the genetic tools described in this study were developed for use in *C. trachomatis* serovar L2 LGV/434. For clarity and consistency in the field, Ct (serovar D) nomenclature is used, but CTL (serovar L2) nomenclature is also referenced where appropriate.

### Plasmid construction

Sequences for primers and crRNAs are provided in Table S1. For the knockdown strains, the vector pBOMBL12CRia::L2 (pBOMBL12CRia) (53) was modified by BamHI-digest, treated with alkaline phosphatase, and the appropriate crRNA gBlock (IDTDNA, Coralville, IA) was inserted using the NEBuilder HiFi DNA assembly kit (New England Biolabs, Cambridge, MA) according to manufacturer protocols. The resulting plasmids were transformed into chemically competent *Escherichia coli* 10-b using conventional techniques. Plasmids from transformants were isolated by miniprep (Qiagen), screened for the correct plasmid by colony PCR or plasmid digest, and were confirmed by sequencing across the crRNA insert site (Genewiz/Azenta).

For the complementation plasmids, the pBOMBL12CRia(*ct226*) plasmid was modified by SalI-digest and insertion of a gBlock (Integrated DNA Technologies, Coralville, IA) containing a ribosomal binding site, a KpnI digest site, and the epitope tag 3×FLAG. Open reading frames for *ct226*, *ct225*, and *ct224* were amplified from *C. trachomatis* L2 genomic DNA using Phusion DNA polymerase (New England BioLabs, Ipswich, MA) and purified using the QIAquick PCR Purification Kit (Qiagen, Hilden, Germany). Purified PCR products were inserted into KpnI-digested pBOMBL12CRia(*ct226*)-3×FLAG vector as described above. The resulting plasmids were isolated as described above and confirmed by sequencing across the insertion site.

### aTc induction conditions for chlamydial strains

For pBOMB4-*ct226-FLAG* and pBOMBLmT-*ct225-3XFLAG* vectors, expression was induced at 7 hpi with 5 nM and 2 nM aTc, respectively. For all knockdown and complement strains, knockdown and/or complementation was induced using 2 nM aTc. Induction timeline for the strains is as follows: for L2/*ct226* KD strain and the L2/*E.V*. control strain, infected cells were induced at 3 hpi. For strains L2/*ct225* KD and L2/*ct224* KD, samples were induced at time of infection. For all complement strains, inclusions were induced with 2 nM aTc at 7 hpi.

### Antibodies and indirect immunofluorescence

Primary antibodies used in these studies were polyclonal rabbit anti-LRRF1 (Bethyl Laboratories), sheep anti-IncA, rabbit anti-FLI1 (Thermo Fisher), goat anti-MOMP (Meridian), mouse anti-FLAG (Sigma-Aldrich), rabbit anti-FLAG (Sigma-Aldrich), mouse anti-Ct225-GST (gift from Guangming Zhong, University of Texas Health Sciences Center-San Antonio), and mouse anti- AsCpf1 (dCas12; Sigma-Aldrich). Secondary antibodies for indirect immunofluorescence included donkey anti-647, -594, and -488 (Jackson Labs, Bar Harbor, Maine). For indirect immunofluorescence microscopy, antibodies were diluted as indicted in 3% bovine serum albumin in phosphate buffered saline (PBS) and incubated for 1 hour at 37°C with the exception of Rb anti-FLI1. Rb anti-FLI1 (Invitrogen-Thermo Fisher) was incubated for 2 hours at 4°C and rocked overnight at 4°C in Dulbecco's phosphate buffered saline (DPBS). DNA detection was performed using DAPI (4′,6-diamidino-2-phenylindole). Western blots were visualized by secondary antibodies conjugated to IRDye 680 or IRDye 800 (LiCor Biosciences, Lincoln, NE).

### Chlamydial transformation

Chlamydial transformations in this study were performed as described previously (69). Briefly, either HeLa or McCoy cells were used for chlamydial transformation and were seeded in six-well plates at a density of 10^6^ on the day prior. Two micrograms of plasmid was added to purified EBs in a Tris-CaCl_2_ buffer solution and allowed to incubate for 30 min at room temperature. Following incubation, the transformation mix was diluted in Hanks’ balanced salt solution (HBSS; Gibco) and added to one well of the six-well plate. Plates were centrifuged at 400 × *g* for 15 min and media was replaced with DMEM+ 10% fetal bovine serum (FBS) following 15 min of incubation at 37°C at 5% CO_2_. At 8 hpi, penicillin was added at either 1 U/mL or 2 U/mL, and cycloheximide was added at concentration of 1 µg/mL (Millipore Sigma). At 48 hpi, infected cells were harvested and centrifuged at 17,000 × *g* for 30 min at 4°C. Pellets were resuspended in 1 mL of HBSS and centrifuged for 5 min at 400 × *g* at 4°C. The supernatant containing the infectious progeny was added to a fresh monolayer of either HeLa or McCoy cells seeded the day before. This cycle of infection followed by progeny harvest was continued until only wild-type inclusions constitutively expressing GFP from the transformed plasmid were observed. Transformed EBs were collected in 2-sucrose-phosphate for storage at −80°C and future plaque-cloning and expansion.

### IFU assay

IFU assays, or infectious progeny assays, were performed as previously described (70, 71). Briefly, all knockdown and complement strains were used to infect a monolayer of HeLa cells in a 24-well plate. Strains were either induced using aTc or not, as described above. At 24 hpi, infected cells were scraped and vortexed with 1 mm glass beads, and freeze-thawed at −80°C. Lysates were collected and used to infect a new monolayer of HeLa cells. Inclusions were counted and reported as IFUs per milliliter. A paired Student’s *t* analysis was performed to determine significant differences between uninduced and induced conditions for each strain. IFUs per milliliter of three independent experiments were reported.

### Time-course localization of Flightless 1 to the inclusion membrane

HEp2 cells were plated in 24-well plates containing cell culture-treated coverslips and infected with wild-type (WT) *Chlamydia trachomatis* serovar L2 (MOI = 1). Coverslips were fixed with 4% paraformaldehyde at the indicated timepoints post infection and permeabilized with 0.5% Triton X-100. Coverslips were processed for indirect immunofluorescence to visualize endogenous FLI1 (green), chlamydial organisms (MOMP; red), and DNA (DAPI; blue). Images were taken on the Zeiss ApoTome.2 fluorescence microscope at 100× magnification. Representative inclusions at indicated timepoints from three biological replicates are shown.

### Co-immunoprecipitation of Ct226-FLAG following siRNA knockdown of LRRF1 and Flightless 1

Non-targeting siRNA (catalog number SR30004; Origene, Rockville, MD), pooled LRRF1 siRNA (catalog numbers 43450, s229968, and s17599; Ambion Life Technologies), and FLI1 (catalog number L-017506-01-0010; Dharmacon, Lafayette, CO) were used in knockdown experiments. HEp2 cells were seeded in six-well plates at a density of 1 × 10^6^ cells/well, and 24 hours post-plating, cells were transfected with 60 nM the appropriate siRNA using JetPrime Transfection Reagent (Polyplus), according to manufacturer protocols. Twenty-four hours after siRNA treatment, media on the cells was replaced, then 48 hours following siRNA transfection, cells were infected with Ctr L2 Ct226-FLAG strain (MOI = 1) and induced as described above (28). Cell lysates were harvested 24 hpi using radioimmunoprecipitation assay buffer (RIPA) buffer (50 mM Tris-HCl, pH 7.4, 150 nM NaCl, 0.1% SDS, and 0.5% sodium deoxycholate) amended with 1% Triton X-100, 1× HALT Protease Inhibitor Cocktail (Thermo Fisher Scientific, Waltham, MA), 1× universal nuclease (Pierce, Rockford, IL), and 150 µM chlamydial protease-like activity factor (CPAF) inhibitor, clasto-lactacystin β-lactone (Santa Cruz Biotechnology, Dallas, TX). Solubilized lysates were clarified, and protein concentration was determined by protein assay (EZQ Protein Quantification Kit; Life Technologies, Carlsbad, CA). Ct226-FLAG was affinity purified using magnetic FLAG beads (Sigma-Aldrich, St. Louis, MO). Bound proteins were eluted by addition of 500 µg/mL FLAG peptide (Thermo Fisher) in 50 µL of RIPA + 1% Triton X-100. The eluate fraction was combined with equal volume of 2× Laemelli amended with 5% β-mercaptoethanol. Samples were resolved by running on an 8% polyacrylamide gel, transferred to polyvinylidene difluoride membrane (pore size, 0.45 µm; Thermo Fisher), and blotted for FLI1 (144 kDa), LRRF1 (dimer; 160 kDa), and Ct226-FLAG (~19 kDa). Representative western blots from three biological replicates are shown.

### Indirect immunofluorescence microscopy for strain characterization and localization studies

HEp2 cells were plated on coverslips placed in a 24-well plate with three technical replicate wells to visualize dCas12, FLI1, or LRRF1. Cells infected with the appropriate strain (MOI = 0.8) and induced as described above. At 24 hpi, cells were fixed using 4% paraformaldehyde and permeabilized with 0.5% Triton X-100. Chlamydial organisms (green) were visualized by constitutive GFP expression from the pBOMBL plasmid. Host and chlamydial DNA was visualized by DAPI (blue). dCas12, LRRF1, or FLI1 was visualized in the red. Coverslips were mounted on slides using ProLong Glass Antifade mounting medium (Thermo Fisher) and imaged using a Zeiss ApoTome.2 fluorescence microscope at 100× magnification. Data are representative of three biological replicates.

### RT-qPCR analysis of knockdown and complementation strains

HEp2 cells were plated in six-well plates at a density of 1 × 10^6^ and, 24 hours post-plating, were infected (MOI = 1) with the appropriate strain and induced as described above. RNA was collected at 3, 12, and 24 hpi using Trizol (Invitrogen/Thermo Fisher), and chloroform extraction was performed for RNA isolation. DNA contamination was removed by use of Turbo DNAfree (Ambion/Thermo Fisher) and done in accordance with manufacturer protocols. DNAse-treated RNA was converted to cDNA by incubation of SuperScript III Reverse Transcriptase (Invitrogen/Thermo Fisher) with random nonamers (New England Biolabs, Ipswich, MA). Resulting cDNA was diluted and stored at −80°C. Diluted cDNA was used in equal volume with SYBR green master mix (Applied Biosystems) for qPCR. Transcripts for *ct226, ct225*, *ct224, ct227*, *ct223* and *16*s were quantified using the standard amplification cycle with a melting curve analysis measured by QuantStudio 3 (Applied Biosystems/Thermo Fisher). A complete list of qPCR primer pairs is provided in Table S1. A standard curve of genomic DNA was generated from isolated *C. trachomatis* serovar L2 genomic DNA. Transcripts were normalized to *16*s transcript levels. Graphs are representative of three biological replicates.

### Quantification of knockdown efficiency

Western blots were imaged using an Azure Biosystems c600 Imaging System. Digital JPEG images of merged LRRF1 or FLI1 (800) with GAPDH (680) were opened with ImageJ and split into individual channels. The resulting split images where then inverted, and densitometry was measured of bands of the appropriate molecular weight for each condition, using rectangles of equal size for each individual band. A “blank” band was also measured to subtract the background fluorescence resulting from the imaging process. The raw integrated density of the “blank” was subtracted from each raw integrated density of protein bands to calculate the “corrected integrated density” measurement. For each blot, the highest corrected integrated density measurement received for GAPDH was used to normalize all other corrected GAPDH values. The corrected integrated densities of either LRRF1 or FLI1 were then divided by the GAPDH normalization factor. Knockdown was calculated with corrected and normalized integrated densities using the following equation: 100 − [(LRRF1 or FLI1 siRNA/NT siRNA) * 100)], and results were graphed using GraphPad Prism.

### Quantification of inclusion area

HEp2 cells were infected with the indicated strains (MOI = 1). Cells were fixed at 24 hpi and stained for immunofluorescence as described above. ImageJ was used to quantify inclusion area using the freehand selection tool. The area of 40 inclusions was quantified per condition for each strain, taken from three biological replicates. An ordinary two-way analysis of variance test was used to determine statistical differences.

### Quantification of fluorescence intensity at the chlamydial inclusion membrane for L2/*ct225* KD strain

HEp2 cells were infected with the L2/*ct225* KD strain and processed for indirect immunofluorescence microscopy as described above for the FLI1 and LRRF1 localization studies. Exposure time was maintained for both the uninduced and induced conditions. Quantification of fluorescence was performed using ImageJ. Briefly, the “Free Hand Line” tool was used to manually draw lines around the inclusion membranes on the “merged channel” image. “Line Width” was set to 12 to cover the width of the inclusion membrane. Once lines were drawn for inclusions on the “merged channel” image, the image for the channel visualizing FLI1 or LRRF1 was used to measure raw integrated density. Data were collected for 90 inclusions for uninduced and induced samples. Because smaller inclusions have a smaller surface area, fluorescence intensity of proteins localized at the inclusion is greater than for larger inclusions. To account for this, data were normalized to inclusion perimeter and reported as raw integrated density/inclusion perimeter. An unpaired Student’s *t*-test was performed to determine statistical significance between uninduced and induced inclusions.

### BACTH and β-galactosidase assays

The BACTH assay was used to test interactions between Ct226, Ct225, and Ct224. The basis for this assay is two genes of interest are transcriptionally fused to either the T25 or T18 subunits of *Bordetella pertussis* adenylate cyclase. If the proteins interact, it results in functional reconstitution of cyclic AMP production in DHT1 *E. coli* lacking adenylate cyclase (D*cya*) and drives expression of β-galactosidase under the *lac* promoter. This assay was performed as previously described (21, 72, 73). Briefly, pST25 and pUT18C vectors fused to either IncA, Ct226, Ct225, or Ct224 (Table S1) were co-transformed into chemically competent DHT *E. coli* (D*cya*) and were plated on minimal medium M63 selection plates containing isoproyl beta-d-1 thiogalactopyranoside (IPTG), 40 mg/mL 5-bromo-4-chloro-3-indolyl-β-D-galactopyranoside (X-Gal), 0.04% casein hydrolysate, and 0.2% maltose. Homotypic interactions of IncA were used for the positive control, while PST25 fused to Ct226 co-transformed with the pUT18C empty vector was used as the negative control. Blue colonies were indicative of a positive interaction, and representative plates were imaged (Fig. S7A). For the β-galactosidase assay, random colonies were picked and grown in M63 minimal media prior to permeabilization with chloroform and 0.1% SDS, which extracts the β-galactosidase. The reaction was allowed to proceed for exactly 20 min, at which it was halted by addition of 1 M NaHCO_3_ and 0.1% *o*-nitrophenol-β-galactoside. Absorbance at the 405 nm wavelength was measured by a Tecan plate reader, normalized to optical density (bacterial growth), and β-galactosidase activity reported as relative units.

## Data Availability

Data associated with this project are uploaded to Mendeley Data (doi: 10.17632/5pmhxj7m5m.1).

## SUPPLEMENTAL MATERIAL

Fig. S1 msphere.00473-24-s0001.tif Control for siRNA knockdown of FLI1 or LRRF1 by indirect immunofluorescence.

Supplemental Figure Legends msphere.00473-24-s0002.docx Legends for the supplemental figures.

Fig. S2 msphere.00473-24-s0003.tif Co-immunoprecipitation of FLI1 with Ct226-FLAG and siRNA knockdown of LRRF1 or FLI1.

Fig. S3 msphere.00473-24-s0004.tif Detection of 3×FLAG-tagged protein in complement strains by western blot.

Fig. S4 msphere.00473-24-s0005.tif Ct225 localization in wild-type Ctr L2 and Ctr L2 Ct225-FLAG strain using endogenous Ct225 antibody.

Fig. S5 msphere.00473-24-s0006.tif LRRF1 and FLI1 localization in the L2/*E.V*. strain.

Fig. S6 msphere.00473-24-s0007.tif Confirmation of LRRF1 siRNA knockdown by immunofluorescence and western blot in cells infected with the Ct226-3×FLAG complement strain.

Fig. S7 msphere.00473-24-s0008.tif Interaction of Ct226 with other candidate Incs in the *ct227* gene cluster by BACTH assay.

Table S1 msphere.00473-24-s0009.docx List of primers, plasmids, and miscellaneous reagents.

ASM does not own the copyrights to Supplemental Material that may be linked to, or accessed through, an article. The authors have granted ASM a non-exclusive, world-wide license to publish the Supplemental Material files. Please contact the corresponding author directly for reuse.
