# Supplementary figures and images for: Chlamydia trachomatis Inc Ct226 is vital for FLI1 and LRRF1 recruitment to the chlamydial inclusion
Source: mSphere. 2024 Oct 15;9(11):e00473-24. doi: 10.1128/msphere.00473-24 (PMC11580450; doi:10.1128/msphere.00473-24)

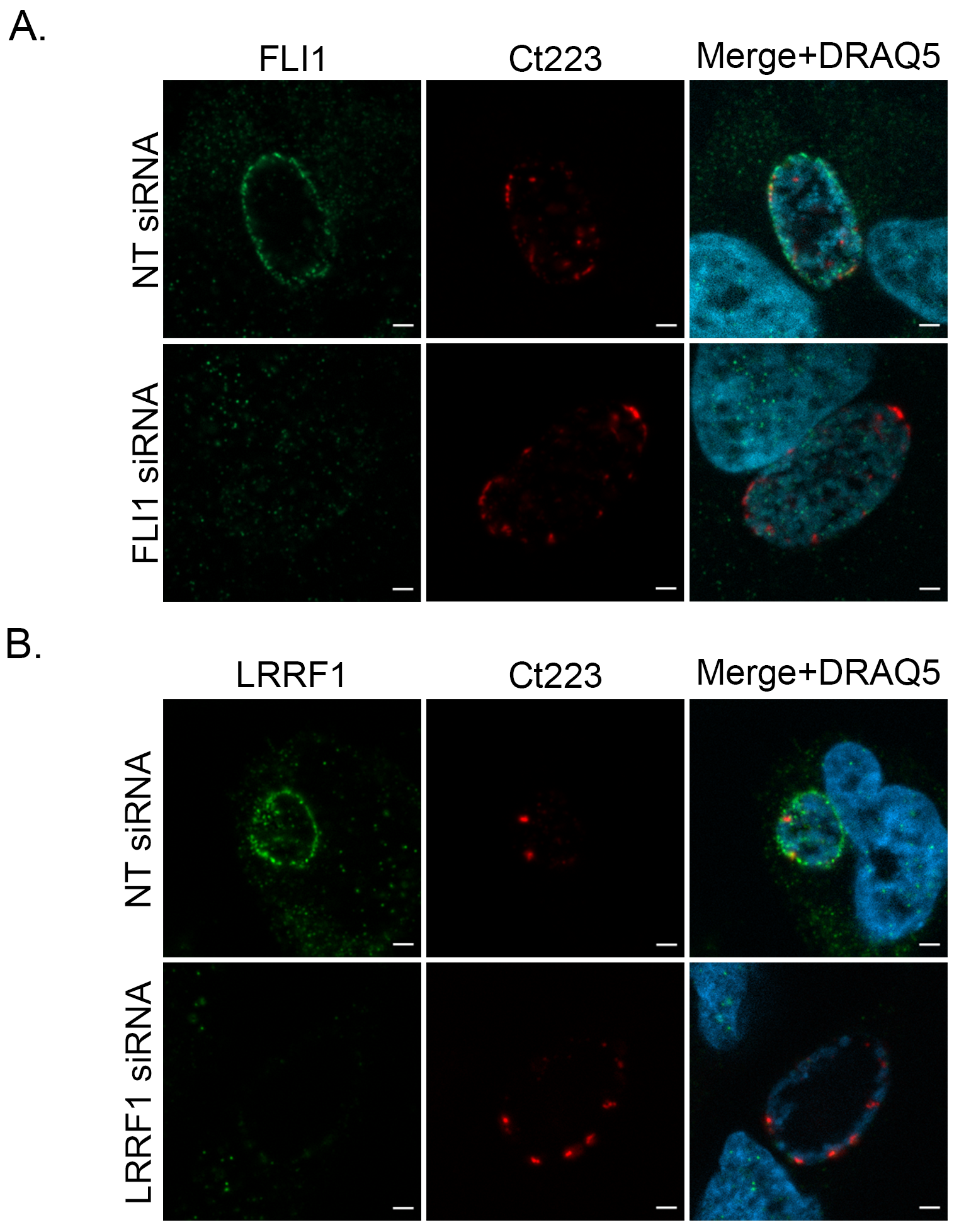

Supplement: Fig. S1 — Control for siRNA knockdown of FLI1 or LRRF1 by indirect immunofluorescence. [file msphere.00473-24-s0001.tif]

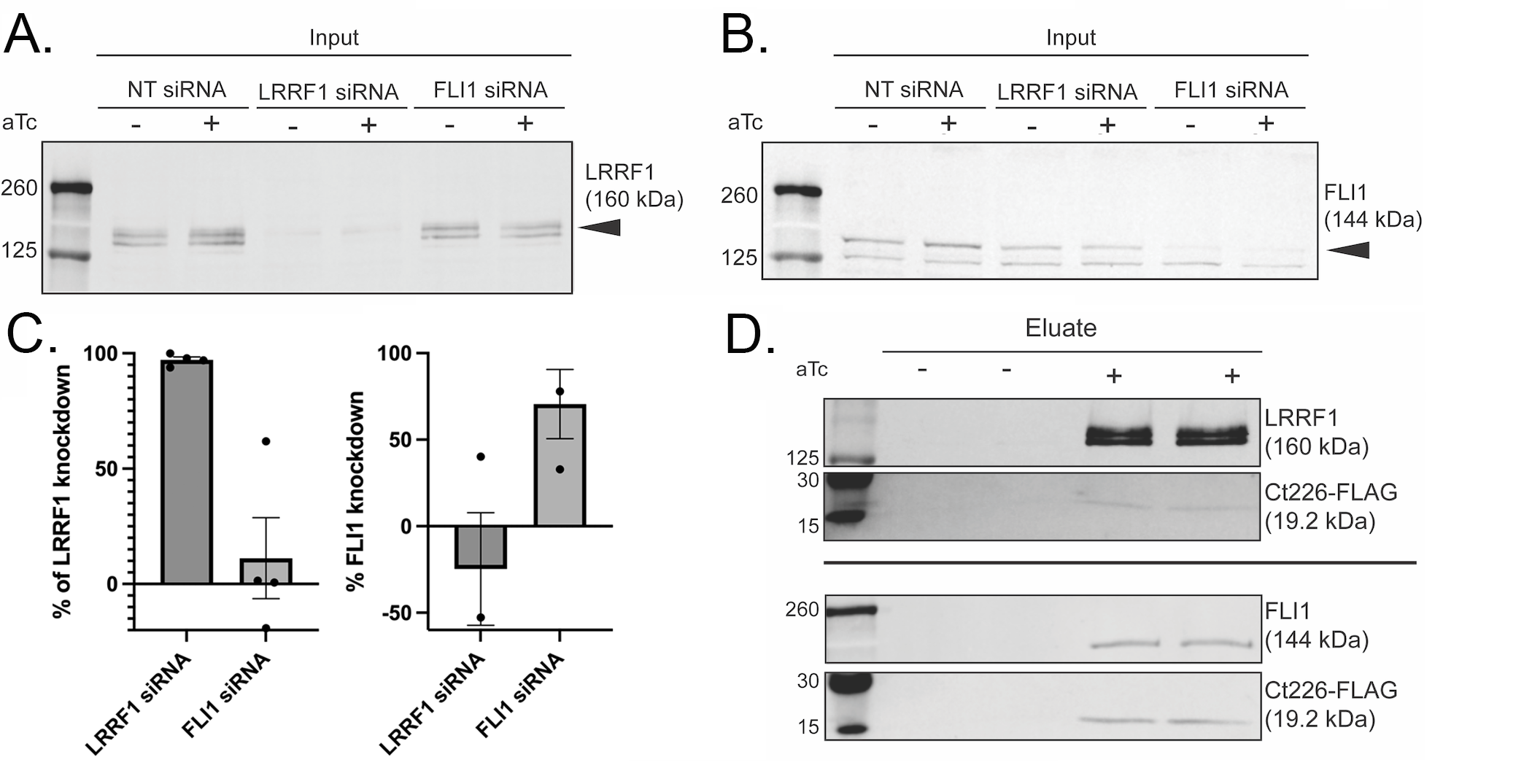

Supplement: Fig. S2 — Co-immunoprecipitation of FLI1 with Ct226-FLAG and siRNA knockdown of LRRF1 or FLI1. [file msphere.00473-24-s0003.tif]

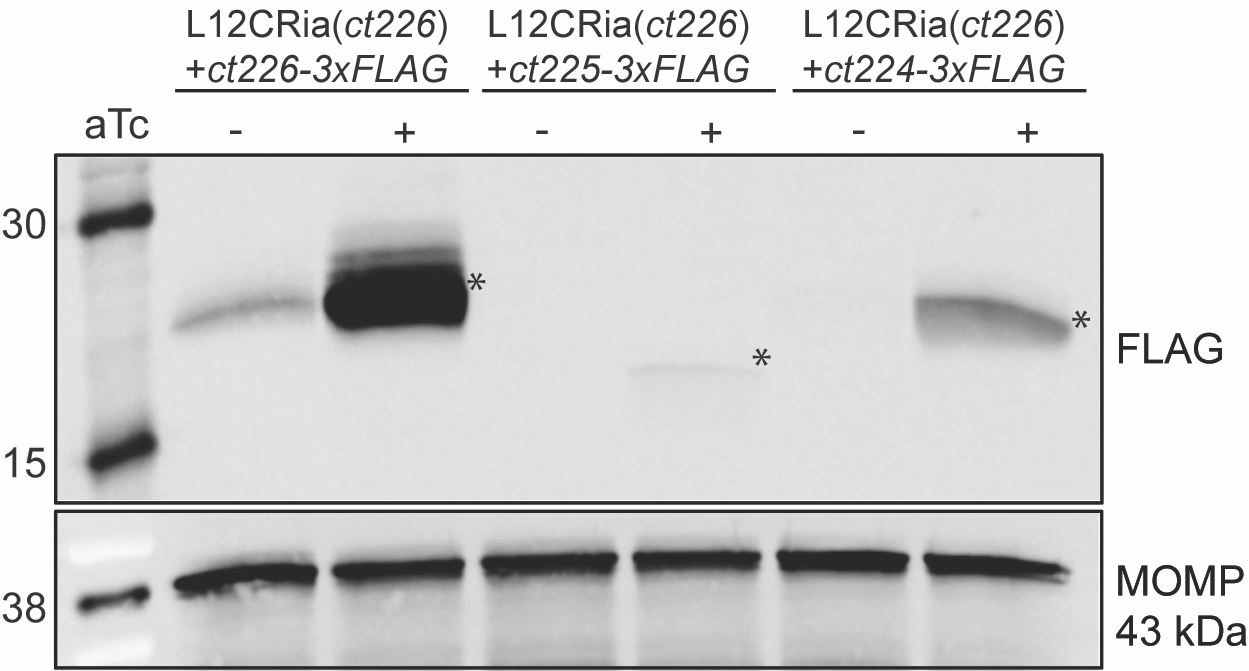

Supplement: Fig. S3 — Detection of 3×FLAG-tagged protein in complement strains by western blot. [file msphere.00473-24-s0004.tif]

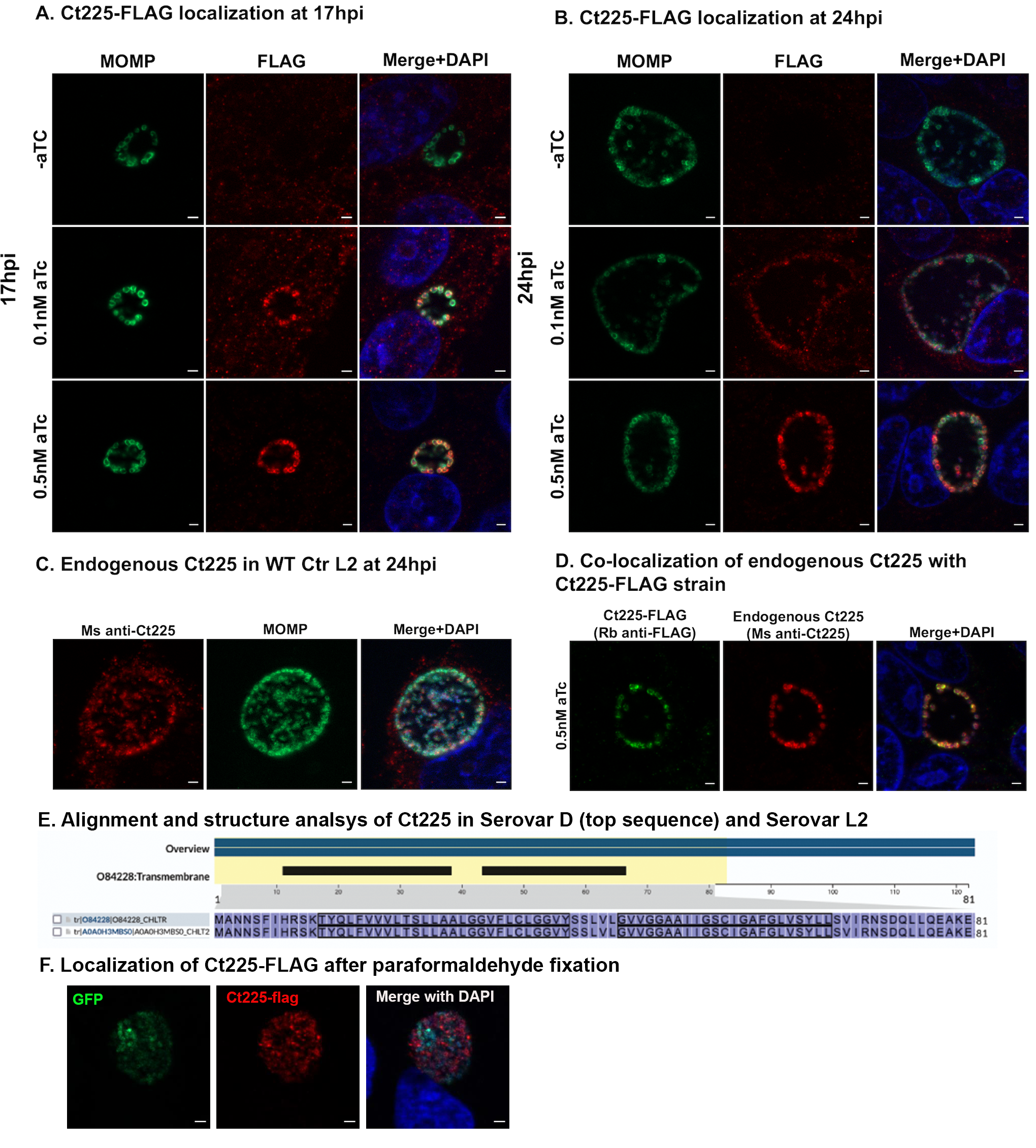

Supplement: Fig. S4 — Ct225 localization in wild-type Ctr L2 and Ctr L2 Ct225-FLAG strain using endogenous Ct225 antibody. [file msphere.00473-24-s0005.tif]

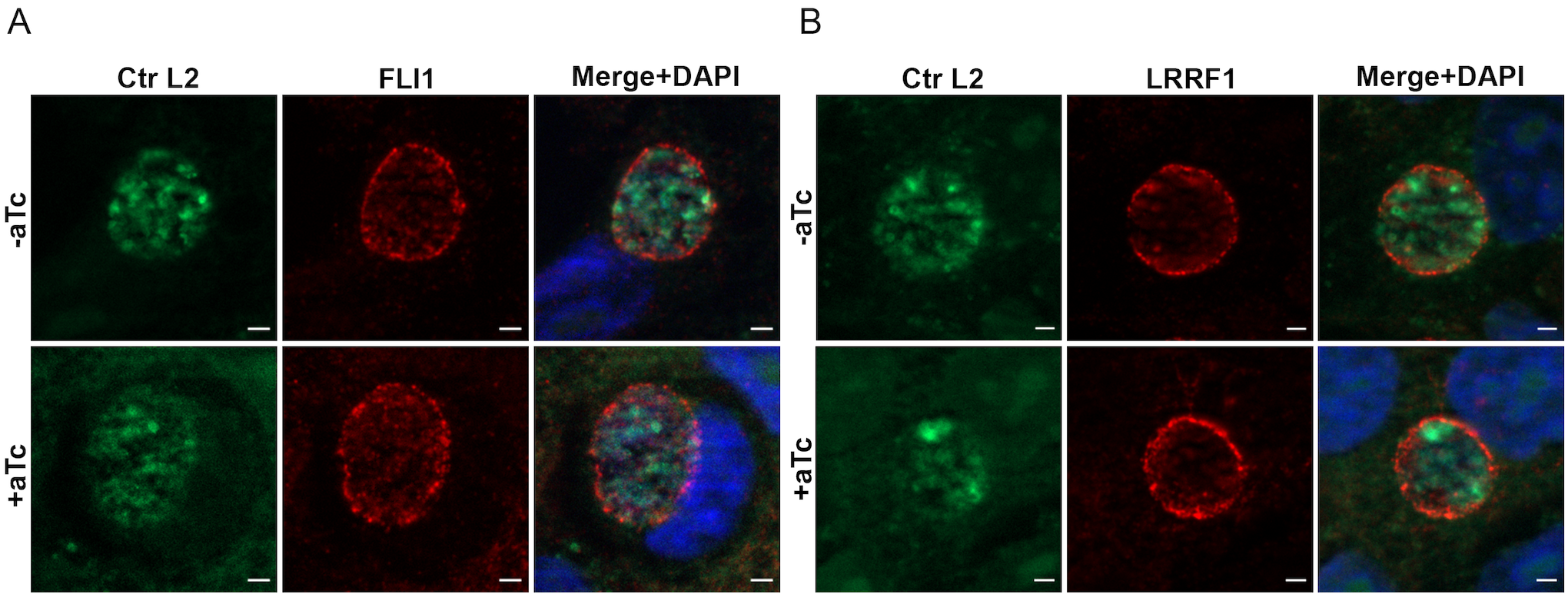

Supplement: Fig. S5 — LRRF1 and FLI1 localization in the L2/E.V. strain. [file msphere.00473-24-s0006.tif]

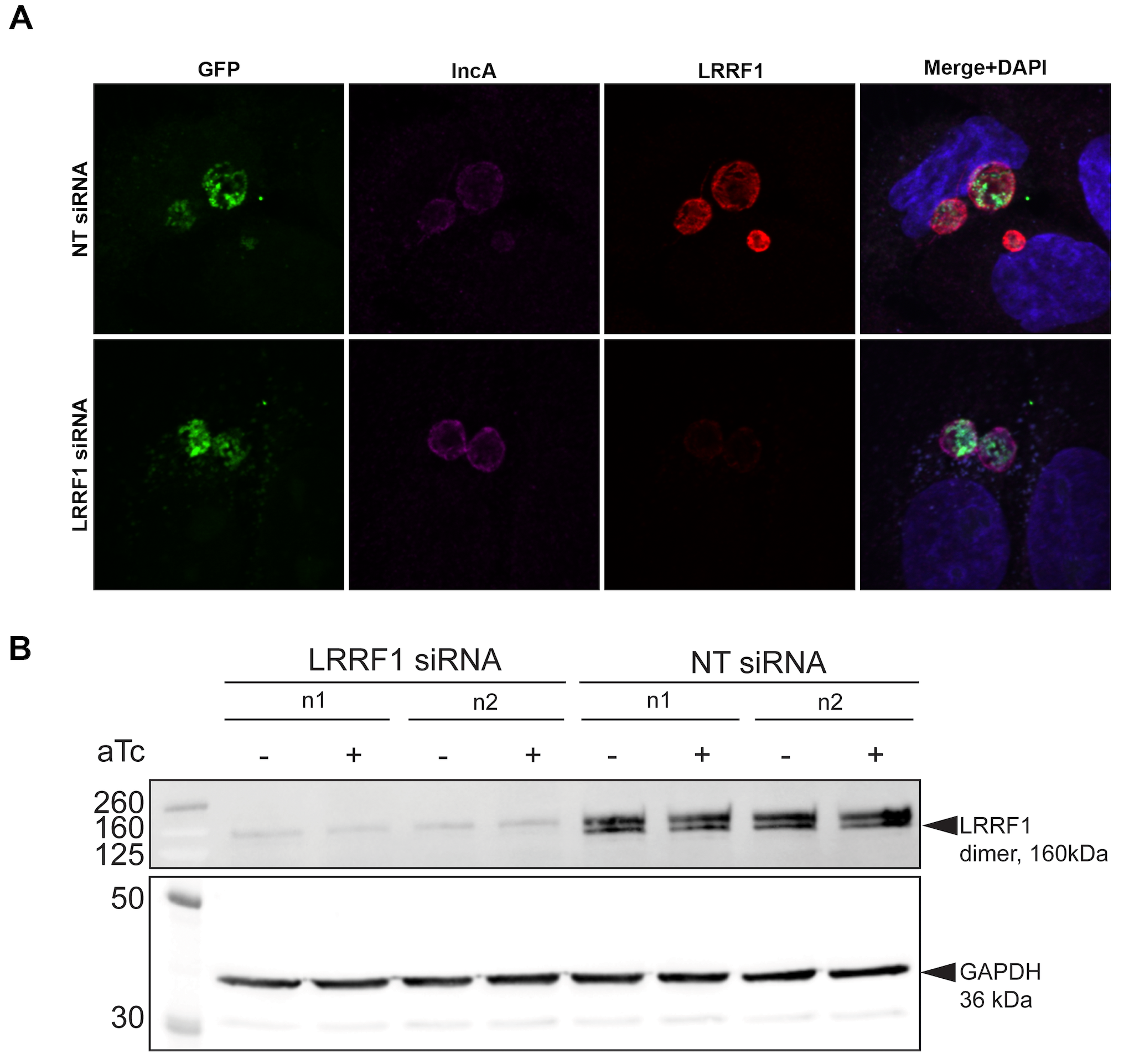

Supplement: Fig. S6 — Confirmation of LRRF1 siRNA knockdown by immunofluorescence and western blot in cells infected with the Ct226-3×FLAG complement strain. [file msphere.00473-24-s0007.tif]

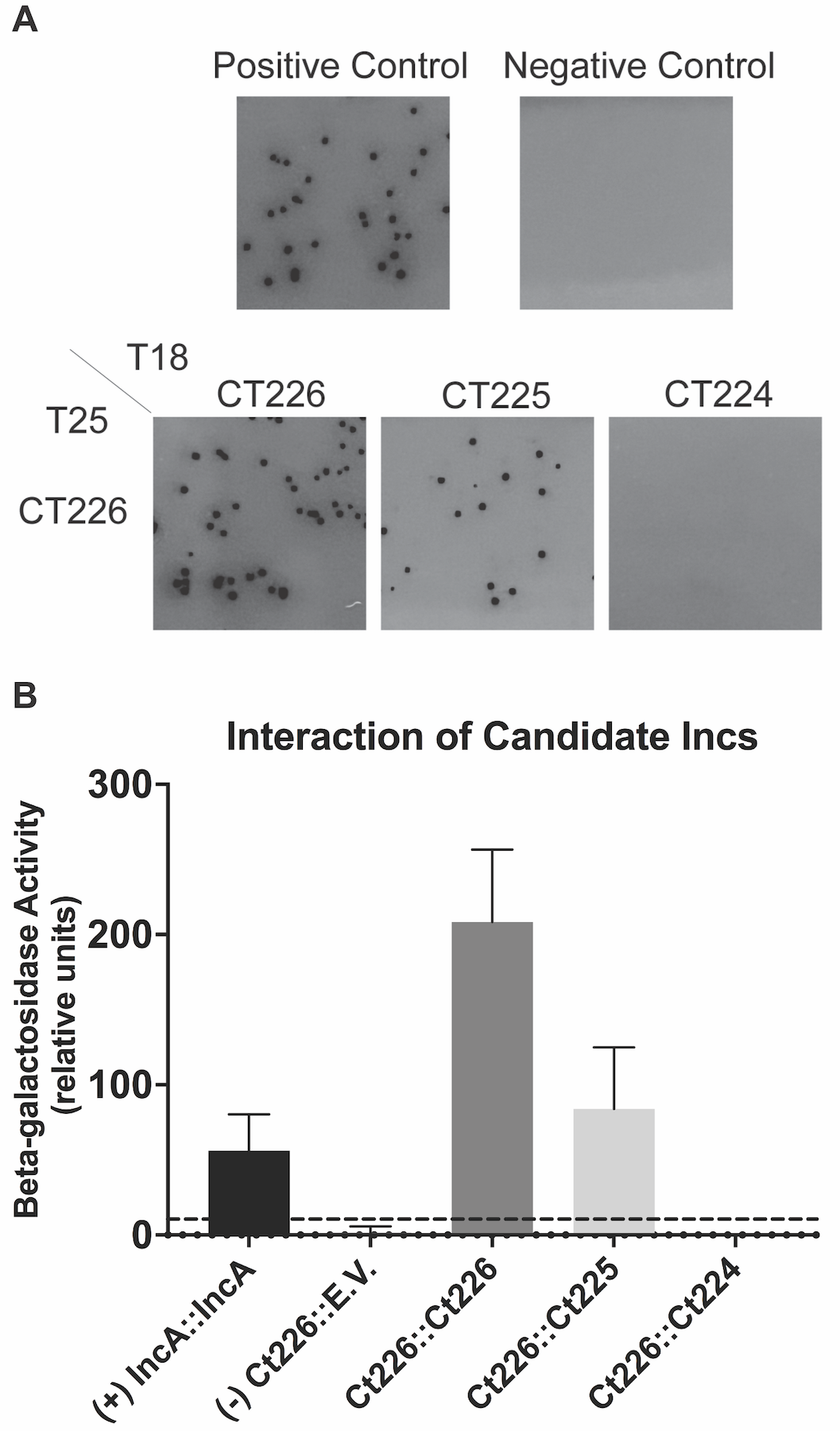

Supplement: Fig. S7 — Interaction of Ct226 with other candidate Incs in the ct227 gene cluster by BACTH assay. [file msphere.00473-24-s0008.tif]
