# Supplementary material for: Chlamydia trachomatis Inc Ct226 is vital for FLI1 and LRRF1 recruitment to the chlamydial inclusion
Source: mSphere. 2024 Oct 15;9(11):e00473-24. doi: 10.1128/msphere.00473-24 (PMC11580450; doi:10.1128/msphere.00473-24)
Supplement: Supplemental Figure Legends — Legends for the supplemental figures. [file msphere.00473-24-s0002.docx]

**Supplemental Figure 1. Control for siRNA knockdown of FLI1 or LRRF1 by indirect immunofluorescence.** Indirect immunofluorescence was used to confirm siRNA knockdown of (A) FLI1 or (B) LRRF1 for Figure 2A. (A) FLI1 visualization at the inclusion membrane following treatment with either non-targeting (NT) siRNA or FLI1 siRNA. (B) LRRF1 visualization at the inclusion membrane following treatment with either NT siRNA or LRRF1 siRNA. Inclusions are representative of three biological replicates.

**Supplemental Figure 2. Co-immunoprecipitation of FLI1 with Ct226-FLAG and siRNA knockdown of LRRF1 or FLI1.** (A-B) Input fractions for Figure 2D. HEp2 cells were treated with NT, LRRF1 or FLI1 targeting siRNA prior to infection with the Ct226-FLAG strain and induced or not with 5nM aTc. Input fractions were collected at 24hpi to assess expression of (A) LRRF1 and (B) FLI1. (C). Knockdown efficiency after transfection with the indicated siRNA from 4 replicates within 3 independent experiments (one experiment had two technical replicates). Calculation of knockdown efficiency is described in Methods and Materials. These results correspond to data reported in **Figure 2C**. (D) Representative western blot of FLI1 co-immunoprecipitation with Ct226-FLAG. HEp2 cells were seeded, infected with the strain carrying pBOMB4-*ct226-FLAG* and induced or not, according to the Materials and Methods. Cell lysates were collected at 24hpi, Ct226-FLAG was affinity purified, eluate fractions were separated by 8% SDS-PAGE gel and transferred to 0.45nm PVDF membrane. The resulting western blot was blotted for FLI1 (anti-FLI1; 144 kDa) and Ct226-FLAG (anti-FLAG; ~19.2 kDa) was used as the loading control. LRRF1 (anti-LRRF1; dimer, 160 kDa) was used a positive control for Ct226-FLAG pulldown. Blot includes two technical replicates and is representative of three biological replicates.

**Supplemental Figure 3. Detection of 3XFLAG-tagged protein in complement strains by western blot.** To assess the expression level of the complemented proteins in each of our complementation strains in both uninduced and induced conditions, protein samples were collected and assessed by western blot. HEp2 cells were plated, infected with one of the complement strains as indicated, and induced as described in the Materials and Methods. Whole cell lysates were blotted with anti-FLAG antibody to detect the complemented protein, as indicated by the asterisk (Ct226-3XFLAG, ~19.2 kDa; Ct225-3XFLAG, ~13-15 kDa; Ct224-3XFLAG, ~16 kDa). Chlamydial MOMP (anti-MOMP; 43 kDa) was used as a loading control.

**Supplemental Figure 4. Ct225 localization in wild-type Ctr L2 and Ctr L2 Ct225-FLAG strain using endogenous Ct225 antibody.** Ectopic expression of Ct225-FLAG at (A) 17 and (B) 24hpi. HEp2 cells were infected with strain carrying pBOMBLmT-Ct225-FLAG and induced with either 0, 0.1, or 0.5 nM aTc at 7hpi. Cells were fixed with methanol at 17hpi and 24hpi and indirect immunofluorescence was used to visualize Ct225-FLAG (anti-FLAG; red), chlamydial organisms (anti-MOMP; green), and host and chlamydial DNA (DAPI; blue). (C) Localization of endogenous Ct225. To determine the localization endogenous Ct225, we used a Ct225 endogenous antibody raised against a Ct225-GST fusion (kind gift of Dr. Guangming Zhong) and used it to localize Ct225 in *C. trachomatis* serovar L2 inclusions (anti-Ct225; red), alongside chlamydial organisms (anti-MOMP; green) and DNA (DAPI; blue). (D) We used the same anti-Ct225 antibody to determine if it recognized the exogenously expressed Ct225-FLAG construct. Indirect immunofluorescence was used to visualize endogenous Ct225 (anti-Ct225; green), Ct225-FLAG (anti-FLAG; red), and DNA (DAPI). Images were taken using a Zeiss ApoTome.2 fluorescence microscope at 100x magnification. Scale bar= 2μm. Inclusions are representative of three biological replicates. (E). FASTA amino acid sequences of Ct225 from Serovar D (O84228_CHLTR Candidate inclusion membrane protein OS=Chlamydia trachomatis (strain D/UW-3/Cx) OX=272561 GN=CT_225; top row) and CTL0477A from Serovar L2 (A0A0H3MBS0_CHLT2 Candidate inclusion membrane protein OS=Chlamydia trachomatis serovar L2 (strain 434/Bu / ATCC VR-902B) OX=471472 GN=CTL0477A; bottom row) were entered into the Uniprot (uniprot.org) protein alignment tools using a Clustal algorithm. The alignment also demonstrated transmembrane domains at amino acids 17-37 and 44-64, consistent with the bilobed transmembrane domain structure of characterized Inc proteins. (F). HEp2 cells were infected as in A and fixed with paraformaldehyde and processed for indirect immunofluorescence and imaged as above.

**Supplemental Figure 5. LRRF1 and FLI1 localization in the L2/*E.V.* strain.** To ensure that expression of dCas12 did not affect LRRF1 and FLI1 localization during chlamydial infection, we used indirect immunofluorescence to localize (A) FLI1 and (B) LRRF1 during infection with the L2/*E.V.* under both uninduced and induced conditions. HEp2 cells were infected with the L2/*E.V.* strain and induced or not at 3hpi. Cells were fixed at 24hpi, and indirect immunofluorescence was used to visualize chlamydial organisms (GFP; green), FLI1 or LRRF1 (anti-FLI1 or anti-LRRF1; red) and DNA (DAPI; blue). Images were taken using a Zeiss ApoTome.2 fluorescence microscope at 100x magnification. Inclusions are representative of three biological replicates. Scale bar= 2μm.

**Supplemental Figure 6. Confirmation of LRRF1 siRNA knockdown by immunofluorescence and western blot in cells infected with the Ct226-3XFLAG complement strain.** HEp2 cells were plated on coverslips and treated with either non-targeting (NT) or LRRF1 siRNA and then infected with the L2/*ct226* KD+*ct226-3XFLAG*. dCas12 and Ct226-3XFLAG expression was induced or not as described in Materials and Methods and cells were fixed at 24hpi by 4% paraformaldehyde. Replicate wells were collected for protein samples to confirm LRRF1 knockdown by western blot. (A) Indirect immunofluorescence was used to visualize chlamydial organisms (GFP; green), the inclusion membrane (anti-IncA; magenta), and LRRF1 (anti-FLI1; red). Host and bacterial DNA were visualized with DAPI (blue). Images were taken on Nikon CSU-W 1 (Nikon, Melville, NY, 541 USA) spinning disk confocal microscope at 60X magnification and are representative inclusions from the induced condition. (B) Western blot detection of LRRF1 in NT and LRRF1 siRNA treated cells. Whole cell lysates were collected at 24hpi, resolved by SDS-PAGE, and transferred to a PVDF membrane for western blotting. The membrane was probed for LRRF1 (anti-LRRF1; 160 kDa dimer) and GAPDH was used as a loading control (anti-GAPDH; 36 kDa). Images and western blots are representative of two biological replicates with two technical replicates each. For B, two technical replicates are indicated by n1 and n2 labeled brackets.

**Supplemental Figure 7. Interaction of Ct226 with other candidate Incs in the *ct227* gene cluster by bacterial adenylate cyclase two-hybrid (BACTH) assay.** To determine if Ct226 can interact with Ct225 or Ct224, we used a bacterial adenylate cyclase two-hybrid (BACTH) assay followed by a beta-galactosidase assay to both qualitatively and quantitatively assess protein-protein interactions. Ct226 fused to the T25 fragment of *Bordetella pertussis* adenylate cyclase was co-transformed with Inc fusions to the T18 fragment adenylate cyclase. IncA homotypic interactions were used as a positive control and co-transfection with the T18-empty vector was used as a negative control. (A) Representative images of BACTH assays testing interactions between Ct226, Ct225, and Ct224. Colony images are representative of three independent experiments. (B) Quantitative analysis of interactions measured by beta-galactosidase assay and reported as relative units for two independent experiments. Values five times the negative control are considered positive.
