# Supplementary material for: Chlamydia trachomatis Inc Ct226 is vital for FLI1 and LRRF1 recruitment to the chlamydial inclusion
Source: mSphere. 2024 Oct 15;9(11):e00473-24. doi: 10.1128/msphere.00473-24 (PMC11580450; doi:10.1128/msphere.00473-24)
Supplement: Table S1 — List of primers, plasmids, and miscellaneous reagents. [file msphere.00473-24-s0009.docx]

Supplemental Table 1: List of Plasmids, Primers, Strains, and gBlocks

| **Plasmid** | **Relevant genotype** | **Ori** | **Source of reference** |
| --- | --- | --- | --- |
| pST25-*ct226* | *aadA* P*_lac_*::*t25-ct226* | p15A | [28] |
| pST25-*incA* | *aadA* P*_lac_*::*t25-incA* | p15A | [28] |
| pUT18C-c*t226* | *bla* P*_lac_*::*t18-ct226* | ColE1 | [28] |
| pUT18C-*ct225* | *bla* P*_lac_*::*t18-ct225* | ColE1 | [21] |
| pUT18C-*ct224* | *bla* P*_lac_*::*t18-ct224* | ColE1 | [21] |
| pUT18C-*incA* | *bla* P*_lac_*::*t18-incA* | ColE1 | [28] |
| pUT18C | *bla* P*_lac_*::*t18* | ColE1 | [73] |
| pBOMBL::L2 | *bla* P_Nm_::*gfp*  P_tet_::*mCherry* | pUC19 | [53] |
| pBOMBLmT::L2 | *bla* P_Nm_::*gfp* | pUC19 | This study |
| pBOMBL(*ct226_FLAG*)::L2 | *bla* P_Nm_::*gfp*  P_tet_::*ct226_FLAG* | pUC19 | [28] |
| pBOMBLmT(*ct225*_FLAG)::L2 | *bla* P_Nm_::*gfp*  P_tet_::*ct225_FLAG* | pUC19 | This study |
| pBOMBL12CRia(*E.V.*)::L2 | *bla* P_Nm_::*gfp* P_tet_::As_dCas12vaa | pUC19 | [53] |
| pBOMBL12CRia(*ct226)*::L2 | *bla* P_Nm_::*gfp* P_tet_::As_dCas12vaa P*_dnaKmut_*::As_crRNA_*ct226* | pUC19 | This study |
| pBOMBL12CRia(*ct225)*::L2 | *bla* P_Nm_::*gfp* P_tet_::As_dCas12vaa P*_dnaKmut_*::As_crRNA_*ct225* | pUC19 | This study |
| pBOMBL12CRia(*ct224)*::L2 | *bla* P_Nm_::*gfp* P_tet_::As_dCas12vaa P*_dnaKmut_*::As_crRNA_*ct224* | pUC19 | This study |
| pBOMBL12CRia(*ct226*)-*ct226-3XFLAG*::L2 | *bla* P_Nm_::*gfp* P_tet_::As_dCas12vaa/*ct226_3XFLAG* P*_dnaKmut_*::As_crRNA_*ct226* | pUC19 | This study |
| pBOMBL12CRia(*ct226*)-*ct225-3XFLAG*::L2 | *bla* P_Nm_::*gfp* P_tet_::As_dCas12vaa/*ct225_3XFLAG*  P*_dnaKmut_*::As_crRNA_*ct226* | pUC19 | This study |
| pBOMBL12CRia(*ct226*)-*ct224-3XFLAG*::L2 | *bla* P_Nm_::*gfp* P_tet_::As_dCas12vaa/*ct224_3XFLAG*  P*_dnaKmut_*::As_crRNA_*ct226* | pUC19 | This study |

| ***E. coli* strain** | **Relevant genotype** | **Source of reference** |
| --- | --- | --- |
| DH5⍺ | *fhuA2 Δ(argF-lacZ)U169 phoA glnV44 Φ80 Δ(lacZ)M15 gyrA96 recA1 relA1 endA1 thi-1 hsdR17* | New England BioLabs |
| DHT1 | F^-^ *glnV44* (AS) *recA1 endA1 gyrA96* (Nal^R^) *thi-1 hsdR17 spoT1 rfbD1 cya-854 ilv-691 ::Tn10 (TetR)* | [73] |
| DH10β | *Δ(ara-leu) 7697 araD139 fhuA ΔlacX74 galK16 galE15 ϕ80dlacZΔM15 (e14-) recA1 relA1 endA1 nupG rpsL (*Str^R^*) rph spoT1 Δ(mrr-hsdRMS-mcrBC)* | New England BioLabs |

| **Primer name** | **Sequence** | **Features** | **Usage** |
| --- | --- | --- | --- |
| pBOMBL12CRia(ct226)_5' Ct226 | actttaagaaggagaggtacATGTTTAATATTTCTTTTTGTTGTAATTC | lower case for plasmid overlap construction | 5' amplification for complementation of Ct226 into pBOMBL12Cria(*ct226)*with 3XFLAG on C-terminus; digest by KpnI |
| pBOMBL12CRia(ct226)_3' Ct226 | tggtctttgtagtctggtacTCTCAGACTTTCTTCCAATAC | lower case for plasmid overlap construction | 3' amplification for complementation of Ct226 into pBOMBL12Cria(*ct226)*with 3XFLAG on C-terminus; digest by KpnI |
| pBOMBL12CRia(ct226)_5' Ct225 | actttaagaaggagaggtacGTGGCTAACAACTCCTTTATTC | lower case for plasmid overlap construction | 5' amplification for complementation of Ct225 into pBOMBL12Cria(*ct226)*with 3XFLAG on C-terminus; digest by KpnI |
| pBOMBL12CRia(ct226)_3' Ct225 | tggtctttgtagtctggtacATCCCACCCATGAAATTTAG | lower case for plasmid overlap construction | 3' amplification for complementation of Ct225 into pBOMBL12Cria(*ct226)*with 3XFLAG on C-terminus; digest by KpnI |
| pBOMBL12CRia(ct226)_5' Ct224 | actttaagaaggagaggtacATGAGTTTTGTTGGAGATAG | lower case for plasmid overlap construction | 5' amplification for complementation of Ct224 into pBOMBL12Cria(*ct226)*with 3XFLAG on C-terminus; digest by KpnI |
| pBOMBL12CRia(ct226)_3' Ct224 | tggtctttgtagtctggtacATCATTGGGAAAAATTGAGTG | lower case for plasmid overlap construction | 3' amplification for complementation of Ct224 into pBOMBL12Cria(*ct226)*with 3XFLAG on C-terminus; digest by KpnI |
| Ct225 Forward | tcttcacacaggacatctgcGTGGCTAACAACTCCTTTATTC | lower case for plasmid overlap construction | For amplification of Ct225 for insertion into the pBOMBLmT vector with FLAG on C-terminus; digest by EagI and NheI |
| Ct225 Rev | tcgtcatccttgtagtcttgATCCCACCCATGAAATTTAG | lower case for plasmid overlap construction | For amplification of Ct225 for insertion into the pBOMBLmT vector with FLAG on C-terminus; digest by EagI and NheI |
| Ct223 qPCR FWD Set 2 | GTTGCTTTGGGAGCTGTTATTT | Forward qPCR primer | For qPCR of *ct223* |
| Ct223 qPCR REV Set 2 | TGAGTTGCTTGTGAGCTTCTAT | Reverse qPCR primer | For qPCR of *ct223* |
| Ct224 qPCR FWD Set 1 | CTTGCGCTCGGTATTCTTAGT | Forward qPCR primer | For qPCR of *ct224* |
| Ct224 qPCR REV Set 1 | CAGGGTAACCACTACGTCAATC | Reverse qPCR primer | For qPCR of *ct224* |
| Ct225 qPCR FWD Set 1 | GTGGGAGGAGCTGCAATTAT | Forward qPCR primer | For qPCR of *ct225* |
| Ct225 qPCR REV Set 1 | GCACTTTCCTTGGCTTCTTG | Reverse qPCR primer | For qPCR of *ct225* |
| Ct226 qPCR FWD Set 1 | CTCAGACTACTACAAGCCGTAAAG | Forward qPCR primer | For qPCR of *ct226* |
| Ct226 qPCR REV Set 1 | GCAGAAGAGCACCACCTAAA | Reverse qPCR primer | For qPCR of *ct226* |
| Ct227 qPCR FWD Set 4 | TGTGTAGCCTTGCTTTGTTTG | Forward qPCR primer | For qPCR of *ct227* |
| Ct227 qPCR REV Set 4 | CCTACTCCTAAAGCCACAGAA | Reverse qPCR primer | For qPCR of *ct227* |

| **gBlock Name** | **Sequence** | **Features** | **Usage** |
| --- | --- | --- | --- |
| *ct226* crRNA | tgtgaaagtgggtcttaagacgtcggtactgcatgtgacgcacgtagatcatgca*TTCACCGGTGGAGACGGTTTTCTTATAATGACACC*TAATTTCTACTCTTGTAGAT**CTTTAATTAGCTTTCCTGTAG**CAAATAAAACGAAAGGCTCAGTCGAAAGACTGGGCCTTTCGTTTTATcaacagcggtctactgaatctgagctagtgcgtgatataattaaaattatattca | Lower case for plasmid overlap and spacer, *italicized* for P_dnaKmut_ promoter sequence, underlined for crRNA scaffold, **bold** for *ct226* targeting sequence, Upper case for rrnB1 terminator | For CRISPRi knockdown of *ct226* |
| *ct225* crRNA | tgtgaaagtgggtcttaagacgtcggtactgcatgtgacgcacgtagatcatgca*TTCACCGGTGGAGACGGTTTTCTTATAATGACACC*TAATTTCTACTCTTGTAGAT**GGGAATTGTTGTTCAGCAAAA**CAAATAAAACGAAAGGCTCAGTCGAAAGACTGGGCCTTTCGTTTTATcaacagcggtctactgaatctgagctagtgcgtgatataattaaaattatattca | Lower case for plasmid overlap and spacer, *italicized* for P_dnaKmut_ promoter sequence, underlined for crRNA scaffold, **bold** for *ct225* targeting sequence, Upper case for rrnB1 terminator | For CRISPRi knockdown of *ct225* |
| *ct224* crRNA | tgtgaaagtgggtcttaagacgtcggtactgcatgtgacgcacgtagatcatgca*TTCACCGGTGGAGACGGTTTTCTTATAATGACACC*TAATTTCTACTCTTGTAGAT**ATAACCGATCTCTTTTACCTT**CAAATAAAACGAAAGGCTCAGTCGAAAGACTGGGCCTTTCGTTTTATcaacagcggtctactgaatctgagctagtgcgtgatataattaaaattatattca | Lower case for plasmid overlap and spacer, *italicized* for P_dnaKmut_ promoter sequence, underlined for crRNA scaffold, **bold** for *ct224* targeting sequence, Upper case for rrnB1 terminator | For CRISPRi knockdown of *ct224* |
| RBS-KpnI-*3XFLAG* | cgtagctgcttaagtaacggTTTGTTTAACTTTAAGAAGGAGAGGTACCA**GACTACAAAGACCATGACGGTGATTATAAAGATCATGACATCGATTACAAGGATGACGATGACAAGTAG**ccattcaaatatgtatccgc | Lower case for plasmid overlap, underlined for the RBS, **bold** for the *3XFLAG* tag | For inserting ribosomal binding site (RBS), KpnI-digest site, and 3XFLAG epitope tag for complementation of tagged Incs downstream of dCas12 coding sequence into the pBOMBL12CRia(*ct226*) vector |
